# Bridging the Implementation Gap: Artificial Intelligence in Periodontology from Proof-of-Concept to Clinical Governance—A Systematic Scoping Review with Evidence from Kazakhstan

**DOI:** 10.3390/dj14090615

**Published:** 2026-09-21

**Authors:** Yerbol Ayash, Aigul Ismailova, Kenesh Dzhusupov, Akerke Chayakova, Anar Aidarkhanova

**Affiliations:** 1 Department of Epidemiology and Biostatistics, Astana Medical University, Astana 010000, Kazakhstan; 202514618@amu.kz (Y.A.); ismailova.a@amu.kz (A.I.); aidarkhanova.a@amu.kz (A.A.); 2 Department of Public Health, International Higher School of Medicine, Bishkek 720054, Kyrgyzstan; k.dzhusupov@ism.edu.kg; 3 Department of Medicine, Osh State University, Osh 723500, Kyrgyzstan

**Keywords:** artificial intelligence, periodontitis, machine learning, deep learning, governance, regulatory framework, Kazakhstan, clinical integration, implementation gap, teledentistry

## Abstract

**Background/Objectives**: Periodontitis, the sixth most prevalent disease worldwide, affects over 740 million people and disproportionately burdens transitional economies. Although artificial intelligence (AI)—including deep learning (DL) and machine learning (ML)—achieves high diagnostic accuracy in research settings, a gap persists between proof-of-concept and real-world deployment, especially where regulation is nascent, as in Kazakhstan. This scoping review maps global evidence on AI for periodontal diagnosis, risk prediction, and monitoring; evaluates governance frameworks; and proposes a contextualised implementation model for emerging health systems. **Methods:** Following PRISMA-ScR and PRISMA 2020 guidance, PubMed/MEDLINE, Scopus, Web of Science, Embase, and Cochrane Library were searched (January 2015–April 2025), supplemented by regulatory and grey literature; ten additional sources published after the search closure were subsequently identified through citation checking and expert peer review during revision, as a targeted amendment rather than a re-executed database search. Forty sources in total (29 peer-reviewed empirical and review studies plus 11 regulatory, grey literature, and patent documents) met the inclusion criteria and were charted thematically. **Results**: Two dominant paradigms emerged: image-based DL (convolutional neural networks and Vision Transformers), achieving 73–98.6% accuracy for radiographic bone loss detection, and ML-based non-clinical screening using patient-reported data and salivary biomarkers. Digital tools (smart toothbrushes, chatbots, and IoT platforms) form a third domain. Performance dropped consistently on external validation, reflecting data quality and sample size constraints. Regulatory analysis showed convergence of the EU AI Act, U.S. FDA framework, WHO guidance, and Kazakhstan’s AI Development Concept (2024–2029) around risk-based classification, transparency, and post-market surveillance. **Conclusions**: Safe, effective AI integration in periodontology requires a phased approach: national multimodal databases, local clinical validation, certified workflow integration, continuous monitoring, population-level surveillance, and legal governance covering liability and insurance. Kazakhstan’s evolving regulatory and digitalisation strategy may offer a context-specific case for evaluating AI adoption pathways across Central Asia and transitional economies, pending prospective validation.

## 1. Introduction

### 1.1. The Global Public Health Burden of Periodontal Disease

Periodontal diseases—encompassing gingivitis and periodontitis—constitute one of the most prevalent and underappreciated chronic non-communicable conditions affecting humanity. Severe periodontitis ranks as the sixth most prevalent disease globally, affecting an estimated 740 million individuals [1]. According to the WHO Global Oral Health Report 2022, oral diseases affect nearly 3.5 billion people worldwide, with periodontal conditions accounting for the largest share of chronic oral disease burden, particularly in low- and middle-income countries (LMICs) [2]. The socioeconomic consequences are profound: untreated periodontitis generates direct treatment costs and substantial indirect productivity losses, with projections indicating a continued upward trajectory as populations age globally [1].

Beyond oral health, periodontitis is bidirectionally associated with systemic conditions including cardiovascular disease, type 2 diabetes mellitus, adverse pregnancy outcomes, and chronic respiratory diseases, amplifying its significance as a systemic risk factor [3]. Despite this magnitude, traditional diagnostic approaches remain fundamentally limited: assessment of periodontal pocket depth, clinical attachment loss, and radiographic bone loss relies on the clinician’s subjective judgement, is inherently invasive, captures historical tissue destruction rather than current disease activity, and is poorly suited to population-level screening [4]. In countries such as Kazakhstan, where dental workforce density is below WHO recommended ratios and geographic access to specialist periodontal care is constrained in rural areas, these diagnostic limitations are especially consequential.

### 1.2. Risk Assessment of Periodontal Disease Based on Determinants

The clinical and public health rationale for AI-assisted periodontal care is grounded in the multifactorial determinant structure of periodontal disease, which makes risk assessment—and not diagnosis alone—central to effective prevention. Periodontal risk is shaped by non-modifiable determinants (age, sex, genetic susceptibility, and ethnicity) and by modifiable behavioural and systemic determinants (tobacco use, glycaemic control in diabetes mellitus, oral hygiene behaviour, socioeconomic status, and access to care). Established risk assessment instruments—including the Periodontal Risk Assessment (PRA) model, the PreViser/Periodontal Risk Calculator, and the Dentist Risk Score—operationalise these determinants into stratified risk categories that guide recall intervals and the intensity of preventive intervention. Crucially, these determinants can be captured as structured, machine-readable variables (clinical charting, patient-reported behaviour, systemic comorbidity, and salivary biomarkers), which is precisely what renders periodontal risk prediction amenable to machine learning approaches. Determinant-based risk stratification thus provides the conceptual bridge between the epidemiological burden described above and the predictive modelling evidence synthesised in Section 3.3, and it directly motivates the population-level risk assessment objective and the community surveillance stage developed later in this review.

### 1.3. The Emergence of Artificial Intelligence in Periodontology: State of the Art

The convergence of digital imaging technology, large-scale clinical data repositories, and advances in computational power has enabled rapid development of AI applications in dentistry since 2015 [5]. Within periodontology, two principal methodological paradigms have emerged. The first is image-based deep learning, wherein convolutional neural networks (CNNs) and attention-based architectures such as Vision Transformers (ViTs) are trained to detect and quantify disease features from radiographic and photographic data. The second is clinical predictive modelling, wherein classical machine learning algorithms—including random forests, gradient boosting, and support vector machines—generate probabilistic risk scores from structured clinical and patient-reported data [6,7]. Recent systematic and narrative reviews have consolidated this rapidly expanding evidence base: AI-powered innovations now span the full diagnostic pathway in periodontology [8], and dedicated systematic reviews have appraised the performance of AI models for the diagnosis, classification, and prediction of periodontal diseases [9], and specifically for distinguishing gingivitis and periodontal disease [10], confirming both the diagnostic promise and the persistent methodological heterogeneity of the field.

Several influential studies have defined the current state of the art. Krois et al. (2019) reported that a seven-layer CNN trained on 2001 panoramic radiograph segments achieved a mean classification accuracy of 0.81 for periodontal bone loss (PBL) detection, which was comparable to the mean accuracy of six experienced dentists (0.76), establishing the foundational proof that CNNs can match clinical expertise in radiographic periodontal assessment [11]. Dujic et al. (2023) extended this work using Vision Transformer networks on periapical radiographs, reporting overall diagnostic accuracy values of 83.4–85.2% and area under the ROC curve (AUC) values of 0.899–0.918, with superior performance for lower anterior teeth, demonstrating that transformer architectures offer a viable alternative to CNNs with improved attention to spatial context [12]. In the domain of multi-disease diagnosis, Kim et al. (2022) developed a web-based AI service employing Faster R-CNN, Inception, and ResNet architectures to simultaneously diagnose five oral pathologies on panoramic radiographs in a Korean hospital setting [13]. Their AI service achieved clinically deployable real-time performance, with each disease type requiring a separately optimised model [13].

For gingival inflammation assessment, Li et al. (2025) proposed GC-U-Net, a deep learning model integrating intraoral scanning (IOS) with global context attention mechanisms, which achieved strong correlations with the Sulcus Bleeding Index (r = 0.836) and the Bleeding Index (r = 0.618), providing a non-invasive, quantifiable alternative to clinical gingival scoring [14]. Extending this multimodal direction to the core diagnostic act itself, Tan et al. (2025) developed PerioAI, a full-stack system that fuses intraoral scanning (IOS) with cone beam computed tomography (CBCT) to segment soft and hard tissues, register them in three dimensions, and directly compute the gingiva–bone distance; evaluated on multicentre cohorts comprising 2507 patients, its digital probing achieved sub-millimetre precision (mean error 0.040 mm), yielding automated, tooth-level, volumetric three-dimensional diagnoses [15]. As the accompanying commentary observes, such IOS–CBCT multimodal pipelines may, pending independent validation, reduce reliance on operator-dependent manual probing and, by quantifying the true extent of periodontal tissue destruction non-invasively, extend the diagnostic information available beyond what conventional probing alone captures [16]. In orthodontic populations, Alalharith et al. (2020) applied Faster R-CNN with ResNet-50 to detect early gingivitis in patients with orthodontic brackets, achieving 77.12% overall accuracy and demonstrating the applicability of deep learning to preventive periodontal monitoring in high-risk subgroups [17].

In predictive modelling, Bashir et al. (2022) conducted a rigorous comparative study of ten machine learning algorithms using nationally representative datasets from the United States and Taiwan, finding that while models achieved high internal validation accuracy, external validation performance dropped significantly—a critical finding exposing the generalisability limitations of AI models trained on geographically and demographically constrained datasets [18]. Deng et al. (2024) demonstrated that ML models combining non-clinical patient-reported parameters with salivary matrix metalloproteinase-8 (MMP-8) and haemoglobin biomarkers from an oral rinse could discriminate between periodontal health, gingivitis, and different stages of periodontitis, opening a pathway for non-invasive population-level screening [19]. The IoT domain has also advanced, with Tonetti et al. (2020) reporting that self-reported bleeding on brushing collected through intelligent power-driven toothbrushes connected to a mobile platform was associated with subsequent bleeding on probing [20]. This was an observational monitoring study analysed with generalised mixed-effects and stepwise regression, not a trained machine learning model; it is therefore treated as contextual IoT evidence rather than AI/ML clinical performance evidence.

A systematic meta-analysis by Khubrani et al. (2025), employing the APPRAISE-AI tool across 2D dental radiographic AI studies from 1990 to 2024, confirmed pooled high sensitivity but noted marked heterogeneity in study design, dataset size, and reporting standards, underscoring the need for standardised methodological frameworks such as MI-CLAIM, CONSORT-AI, and SPIRIT-AI to ensure reproducibility and clinical translatability [21]. Ferrara et al. (2025) similarly highlighted through QUADAS-2 quality assessment that deep learning models in periodontology achieve accuracies ranging from 73.0% to 98.6% in controlled settings, but that most studies lack external validation, and that real-world clinical deployment remains nascent [7].

### 1.4. Research Gap: From Proof-of-Concept to Clinical Governance

Despite this rapidly expanding body of technical evidence, a fundamental implementation gap persists. The vast majority of AI periodontology studies are single-centre, conducted in high-income countries (primarily the United States, South Korea, China, Germany, and Japan), and validated on demographically narrow datasets that do not represent the global population. The AI-in-dentistry-in-LMICs scoping review by Umer et al. (2024) found only 25 studies from lower-middle-income countries despite an initial yield of 1587 records, confirming the severe underrepresentation of transitional economies in this research domain [22]. This geographic concentration creates a paradox: the countries with the greatest unmet need for diagnostic support have the least applicable evidence base.

Equally critical is the regulatory dimension. The EU Artificial Intelligence Act (Regulation 2024/1689) classifies AI-based medical diagnostic systems as high-risk, imposing mandatory conformity assessments, clinical validation, algorithmic transparency, and continuous post-market surveillance [23], while the U.S. FDA’s Software as a Medical Device (SaMD) framework establishes a risk-tiered pre-market pathway with algorithmic change protocols for adaptive AI [24]. Transitional economies including Kazakhstan have, until recently, lacked operationalised equivalents of these frameworks, although Kazakhstan has now enacted a dedicated AI Law (Section 3.8); the downstream consequences of this historical gap for liability, clinical adoption, and insurance coverage are examined in Section 4.1 (Discussion) rather than restated here.

Kazakhstan presents a relevant and underexplored case study. As a rapidly modernising upper-middle-income country with an ambitious Digital Kazakhstan national program, a newly published AI Development Concept for 2024–2029 (Resolution No. 592), and a healthcare system undergoing significant digitalisation, Kazakhstan occupies a position of strategic relevance for the broader Central Asia and post-Soviet regional health policy context [25]. Yet no comprehensive analysis has assessed Kazakhstan’s specific regulatory readiness, clinical infrastructure capacity, and translational pathway for AI integration in periodontal care.

Three recent evidence syntheses define the current state of the art and delimit what a further review must add. Scott et al. (2023) mapped 50 studies of AI in periodontology and implantology and concluded that reporting outcomes are too heterogeneous to permit meaningful comparison, that no included study benchmarked a model against a genuinely independent examiner on unseen data, and that research output is concentrated in the United States, China, and South Korea [6]. Chatzopoulos et al. (2025) charted 66 studies across seven clinical application domains—alveolar bone loss, intrabony and furcation defects, gingivitis, biofilm and calculus, disease staging, diagnostic communication, and miscellaneous applications—and reported diagnostic performance frequently matching or exceeding that of dental professionals, while identifying dataset size and diversity as the principal outstanding constraint [26]. Roy et al. (2025) extended this synthesis narratively to peri-implant as well as periodontal diagnosis, covering imaging, immunological biomarkers, and microbial profiles [27]. Taken together, these reviews establish, with considerable authority, what AI can do in periodontology under research conditions. None of them addresses the question that determines whether any of it reaches a patient: the conditions under which such models can be lawfully certified, locally validated, reimbursed, and monitored within a specific health system. All three are organised by clinical task and confine implementation to a closing call for larger and more diverse datasets; none analyses regulatory instruments, liability allocation, data governance infrastructure, or workforce and reimbursement constraints, and none examines a transitional health system. The present review is therefore positioned deliberately downstream of this literature. It takes the diagnostic performance evidence largely as established by these syntheses, and treats governance and implementation—rather than model accuracy—as the primary analytical object, using Kazakhstan as a worked case.

### 1.5. Research Objectives

In light of these gaps, the present systematic scoping review was designed to address three interconnected research objectives:

1. To systematically map and synthesise the methodological evidence base for AI applications in periodontal diagnosis, determinant-based and population-level risk prediction, and patient monitoring, with specific attention to the methods used, performance metrics, and validation approaches.
2. To evaluate the principal technical, data quality, and clinical barriers constraining the transition of AI tools from proof-of-concept to scalable, real-world clinical deployment, and to analyse and compare international regulatory and governance frameworks (EU, USA, WHO, and UNESCO) and assess their applicability to Kazakhstan’s emerging regulatory context.
3. To develop and propose a contextualised, evidence-based implementation framework—spanning national data infrastructure, local validation, digital workflow integration, continuous monitoring, population-level periodontal risk surveillance, and legal governance—for the safe, ethical, and clinically valid integration of AI into periodontal care in Kazakhstan and analogous transitional health systems.

These objectives distinguish the present work from prior reviews—which have largely focused on technological performance metrics—by centring governance, regulatory readiness, population-level risk assessment, and implementation feasibility as primary analytical dimensions. Consistent with this framing, the primary contribution of the manuscript is the proposed utilisation and governance structure for Kazakhstan; the global evidence synthesis provides the evidential basis for that structure.

## 2. Materials and Methods

### 2.1. Study Design and Methodological Justification

This study was conducted as a systematic scoping review following the Preferred Reporting Items for Systematic Reviews and Meta-Analyses extension for Scoping Reviews (PRISMA-ScR) and the PRISMA 2020 reporting framework [28,29]. Throughout, the work is reported as a systematic scoping review; no quantitative pooling, meta-analysis, or conventional systematic review effect estimation was performed, and the term ‘systematic review’ is used only when referring to other authors’ cited studies. A completed PRISMA 2020 27-item checklist [30] and a PRISMA-ScR 20-item checklist documenting compliance with each reporting item, together with the location of the relevant information in the manuscript, are provided in Supplementary Materials S1 and S2. A scoping review design was selected—rather than a conventional systematic review with meta-analysis—for several evidence-based reasons. First, the clinical outcomes of AI in periodontology are highly heterogeneous in terms of AI model types, input data modalities (radiographic, photographic, biomarker, and patient-reported), outcome measures, and performance metrics, precluding meaningful statistical pooling. This heterogeneity was confirmed by prior meta-analyses including the study by Khubrani et al. (2025), which reported marked I^2^ statistics necessitating sensitivity analyses even within narrowly defined sub-questions [21]. Second, the review explicitly encompasses non-empirical evidence—including regulatory documents, policy frameworks, governance guidelines, and technical specifications—which constitutes a substantial and scientifically necessary component of the research objectives and is incompatible with conventional systematic review methodologies. Third, the PRISMA-ScR framework is specifically recommended by the Joanna Briggs Institute for multi-domain evidence synthesis where mapping breadth and coverage are prioritised over depth of a single outcome [29].

The methodological choice to employ thematic synthesis rather than quantitative meta-analysis was further justified by the consistent finding across recent reviews that AI periodontology studies are methodologically too diverse for reliable pooling: Ferrara et al. (2025) included only 13 studies meeting QUADAS-2 quality criteria from a broad search [7], while Scott et al. (2023) found reporting outcomes ‘heterogeneous because of the variety of statistical tests available’ across 50 included studies [6]. Thematic synthesis enables identification of convergent and divergent evidence patterns across these heterogeneous study designs, providing a more valid basis for regulatory and implementation recommendations than would be possible through forced quantitative pooling.

### 2.2. Protocol and Registration

An a priori protocol specifying the review question, eligibility criteria, information sources, search strategy, study selection process, data-charting approach, and synthesis plan was developed by the authorial team before commencing the database searches. Because PROSPERO does not accept scoping reviews, the protocol was not prospectively registered in PROSPERO; instead, it was documented internally at Astana Medical University’s Department of Epidemiology and Biostatistics and is available from the corresponding author upon reasonable request. Two amendments to the protocol are recorded. First, minor refinements to the search strings (i.e., the addition of ‘convolutional neural network’, ‘natural language processing’, and ‘vision transformer’ as AI synonyms after pilot searching) were implemented. Second, after the search closure date of 30 April 2025, ten additional sources meeting the pre-specified eligibility criteria but published after this date were identified through expert peer review, citation checking, and reviewer requests during the revision process, rather than through a repeat execution of the five-database and patent search; both amendments are documented in Supplementary Material S3. Of the ten sources added under the second amendment, six are peer-reviewed empirical, review, or interpretive studies—Jafer et al. (2025) [9], Tan et al. (2025) [15], Wu & Tsoi (2025) [16], Yan et al. (2025) [31], Issilbayeva et al. (2025) [32], and Sarakbi et al. (2025) [33]—incorporated into the formal qualitative synthesis on the same eligibility basis as the originally identified sources. Four sources are regulatory, grey literature, or patent documents—granted Russian patents RU2841194 [34] and RU2850170 [35], Kazakhstan’s enacted AI Law [36], and the national AI supercomputer infrastructure source [37]—added specifically to update the regulatory and Kazakhstan context analysis (Section 3.6 and Section 3.8); in the case of the two patents, both had been provisionally located as pending applications during the original patent search but were reclassified here as revision additions once verification showed that their grant/publication dates (3 June 2025 and 5 November 2025, respectively) postdate the search closure (Supplementary Material S5). This update was a targeted, citation- and peer-review-triggered amendment rather than a full re-execution of the systematic search across all five databases and the patent registry to a single common closure date; consequently, formal completeness of the evidence base beyond 30 April 2025 cannot be claimed, and this scope limitation is stated explicitly in Section 4.4. Full details, including the exact PRISMA counts, are given in Section 3.1.

### 2.3. Eligibility Criteria (Population, Concept, and Context)

Eligibility criteria were structured according to the Joanna Briggs Institute Population–Concept–Context (PCC) framework for scoping reviews [29]:

- Population: Paediatric, adolescent, and adult patient populations with diagnosed or suspected gingivitis, periodontitis, periodontal bone loss, or at population-level risk of periodontal disease, including populations targeted for determinant-based risk stratification and community-level periodontal screening. Regulatory and governance frameworks applicable to AI medical devices used in such populations were also eligible.
- Concept: Development, validation, deployment, or governance of artificial intelligence (AI), machine learning (ML), or deep learning (DL) models for periodontal diagnosis, risk prediction, severity classification, or patient monitoring; or analysis of regulatory, ethical, legal, or implementation frameworks pertaining to such AI tools.
- Context: Global evidence base, with no geographic restriction; particular emphasis on transitional and upper-middle-income health systems (including Kazakhstan, Central Asia, and analogous settings).

Inclusion criteria: Sources were eligible if they: (1) applied AI, ML, or DL methods to periodontal diagnosis, prediction, classification, or patient monitoring; (2) used clinical imaging data (intraoral photographs, panoramic radiographs, periapical radiographs, and CBCT), patient-reported outcomes, biomarkers, or digital device data as input; (3) reported quantitative performance metrics or qualitative implementation outcomes; (4) were peer-reviewed original studies, systematic reviews, or scoping reviews; or (5) constituted authoritative regulatory, policy, or clinical governance documents relevant to AI in healthcare. Eligible publication types included original research articles, systematic reviews, scoping reviews, meta-analyses, regulatory texts, official policy documents, and granted patents. The time window for the original systematic search was 1 January 2015 to 30 April 2025; ten sources published after this date were subsequently admitted under a separately documented, explicitly labelled targeted supplementary update (Section 2.2) rather than under an extended systematic search window, and this distinction is maintained throughout Section 2 and Section 3 (Materials and Methods, Results). Eligible languages were English and Russian.

In addition to primary studies directly applying AI/ML/DL methods, the following pre-specified contextual categories were also eligible, given their function as evidence map, methodological, or population context sources rather than as primary clinical performance evidence: (i) systematic or scoping reviews and meta-analyses synthesising AI applications in periodontology, used for state-of-the-art positioning and evidence gap identification (e.g., [6,8,9,10,21,33,38]); (ii) a single companion commentary directly interpreting an included primary study (Wu & Tsoi 2025 [16], interpreting Tan et al. 2025 [15]); (iii) a non-AI diagnostic accuracy or biomarker validation study directly underpinning the input modality of an included AI/ML screening study (Deng et al. 2022 [39], validating the point-of-care biomarker assay used in Deng et al. 2024 [19]); (iv) a methodological/reporting standards source used to benchmark AI-specific reporting quality (Gusev et al. 2022 [40]); (v) non-AI teledentistry systematic review protocols cited for their relevance to the IoT/monitoring theme and Stage 4 of the proposed implementation framework, though they do not themselves evaluate AI/ML methods (Kengne Talla et al. 2023 [41], 2024 [42]); and (vi) Kazakhstan-specific epidemiological or microbiome sources cited to establish local population context rather than AI evidence (Sagatov et al. 2022 [43]; Issilbayeva et al. 2025 [32]). These contextual sources are charted in Supplementary Table S4 and Group A of Supplementary Material S5 alongside primary AI studies but are explicitly distinguished from primary clinical performance evidence in the stratified synthesis described in Section 2.10 and in Supplementary Table S6, and are not used to support claims about AI diagnostic or predictive performance.

Exclusion criteria: Sources were excluded if they: lacked a clear description of AI methodology; focused exclusively on non-periodontal dental applications without relevant comparative data; were published in languages other than English or Russian without an available translation; represented duplicate analyses of the same dataset; or were opinion pieces, editorials, letters, or commentaries without primary evidence. As a single, pre-specified exception to this last criterion—i.e., the protocol specified in advance, before the search, that a companion commentary directly linked to an included primary study would be eligible when used solely for interpretive synthesis of that study—one commentary meeting this rule was identified: Wu & Tsoi (2025) [16], accompanying the PerioAI study [15]. Because this commentary was itself published after the 30 April 2025 search closure, the qualifying instance was identified during the post-closure revision update rather than the original search (Section 2.2); this affects only the timing of discovery, not the pre-specification of the eligibility rule itself. For editorial audit, the internally documented a priori protocol (Section 2.2), which records this exception, should be retained and made available upon request, consistent with the existing data availability practice for this review.

### 2.4. Information Sources

The search was conducted in five major international academic databases: (1) PubMed/MEDLINE; (2) Scopus; (3) Web of Science Core Collection (Science Citation Index Expanded, Social Sciences Citation Index, and Emerging Sources Citation Index); (4) Embase (Elsevier); and (5) Cochrane Central Register of Controlled Trials and the Cochrane Database of Systematic Reviews. To capture patents and technical solutions, WIPO PatentScope was additionally searched. Grey literature—including international guidelines, regulatory documents, and national policy frameworks—was retrieved from the official websites of the World Health Organization (WHO), the U.S. Food and Drug Administration (FDA), the European Union legislative database (EUR-Lex), UNESCO, the International Medical Device Regulators Forum (IMDRF), and the national legal database of the Republic of Kazakhstan (adilet.zan.kz). Backward citation searching of all included sources was performed to identify additional relevant publications not captured by the database queries. The last search was executed on 30 April 2025.

### 2.5. Search Strategy

Search strategies were developed iteratively by two reviewers (A.C. and A.A.) with input from a senior methodologist (Y.A.) and refined through pilot searching of PubMed/MEDLINE before adaptation to each database. The strategy combined three conceptual blocks linked with the Boolean operator AND, with synonyms within each block linked with OR: (1) *AI technology terms:* ‘artificial intelligence’ OR ‘machine learning’ OR ‘deep learning’ OR ‘convolutional neural network’ OR ‘neural network’ OR ‘natural language processing’ OR ‘vision transformer’; (2) *periodontal disease terms:* ‘periodontitis’ OR ‘periodontal disease’ OR ‘gingivitis’ OR ‘periodontal bone loss’ OR ‘alveolar bone loss’ OR ‘gingival inflammation’; and (3) *contextual terms* applied where appropriate: ‘diagnosis’ OR ‘screening’ OR ‘prediction’ OR ‘monitoring’ OR ‘classification’ OR ‘governance’ OR ‘regulation’. Database-specific MeSH (PubMed/MEDLINE), Emtree (Embase), and free-text fields were used; truncation (*) and proximity operators were applied where syntax allowed. Filters were applied for publication date (2015–2025) and language (English, Russian). No filters were applied for study design, geography, or document type. The full database-specific search strings, including the precise number of records retrieved from each database, are reported verbatim in Supplementary Material S3 to enable reproducibility.

### 2.6. Selection Process

**Figure 1:**
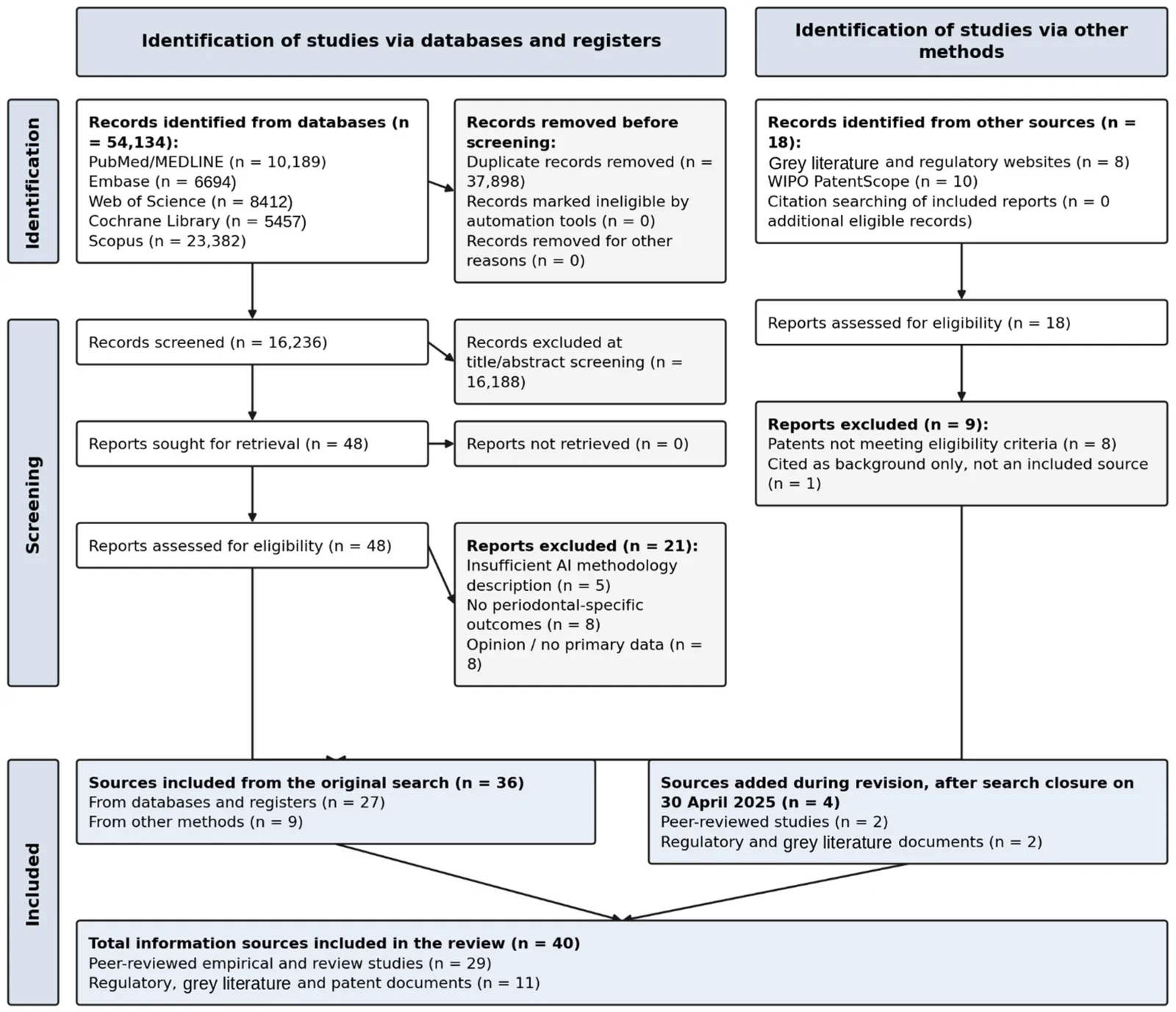
PRISMA 2020 flow diagram of study identification, screening, and inclusion. Records from databases were identified through PubMed/MEDLINE (*n* = 10,189), Embase (*n* = 6694), Web of Science (*n* = 8412), Cochrane (*n* = 5457), and Scopus (*n* = 23,382); after removal of 37,898 duplicate records, 16,236 records were screened at title/abstract, of which 16,192 were excluded, leaving 44 reports assessed for eligibility (all 44 sought were retrieved), of which 21 were excluded and 23 included, reflecting the original search closed 30 April 2025. Records identified through other methods within the original search window (*n* = 16: 8 grey literature/regulatory sources and 8 WIPO PatentScope records not yet granted as of 30 April 2025) were all assessed for eligibility, of which 9 were excluded and 7 (regulatory sources) included, giving 30 sources from the original search (23 databases and registers + 7 other methods). Ten further sources—six peer-reviewed empirical/review/interpretive studies (Jafer et al. 2025 [9]; the PerioAI study [15] and its companion commentary [16]; Yan et al. 2025 [31]; Issilbayeva et al. 2025 [32]; Sarakbi et al. 2025 [33]) and four regulatory/grey literature/patent documents (granted Russian patents RU2841194 [34] and RU2850170 [35]; Kazakhstan’s enacted AI Law [36]; and the national AI supercomputer infrastructure source [37])—were identified through expert peer review and citation checking and added during revision; all ten were published after the 30 April 2025 search closure. The two patents had been provisionally located as pending applications during the original patent search but are reclassified here as revision additions because their verified grant/publication dates (3 June 2025 and 5 November 2025, respectively) postdate search closure. This revision update was a targeted, citation-triggered amendment and not a full re-execution of the database and patent searches (Section 2.2); the database search figures above describe the original search only. Adapted from Page MJ et al. The PRISMA 2020 statement. BMJ 2021;372:n71. doi:10.1136/bmj.n71 [30].

All records retrieved from databases were exported to a reference management database (Zotero, version 6.0) for deduplication. After automated and manual deduplication, the remaining records underwent two-stage screening. In Stage 1, titles and abstracts of all unique records were screened independently and in duplicate by two reviewers (A.C. and A.A.) against the pre-specified eligibility criteria. In Stage 2, full texts of records judged potentially eligible at Stage 1 were retrieved and assessed independently and in duplicate by the same two reviewers. Disagreements at either stage were resolved by consensus discussion; unresolved disagreements were adjudicated by a third reviewer (Y.A.). Inter-rater agreement at Stage 1 was substantial (Cohen’s κ = 0.81). No automation tools were used to support screening decisions. Reasons for exclusion at the full-text stage were recorded for each excluded report and are summarised in the PRISMA 2020 flow diagram (Figure 1).

### 2.7. Data-Charting (Extraction) Process

A structured data-charting form was developed a priori by the authorial team, calibrated on the first five included studies, and refined to ensure consistency before charting the remaining sources. Data were extracted independently by two reviewers (A.C. and A.A.) using a piloted Microsoft Excel template. Discrepancies in extracted values were resolved by consensus or by referral to a third reviewer (Y.A.). Where reported information was ambiguous or incomplete, corresponding authors were not contacted, given the descriptive (non-quantitative-synthesis) nature of the review; instead, missing items were recorded as ‘not reported’ in the extraction table. No automation tools were used to support data charting.

### 2.8. Data Items

For each included empirical study, the following items were charted: (i) bibliographic details (author, year, country, and journal); (ii) study design and setting; (iii) sample size and characteristics of the training, validation, and (where applicable) external test sets; (iv) AI/ML/DL model type and architecture (e.g., CNN, Faster R-CNN, U-Net, Vision Transformer, random forest, and gradient boosting); (v) input data modality (panoramic radiograph, periapical radiograph, intraoral scan, CBCT, biomarker, patient-reported data, and IoT data); (vi) clinical application domain (radiographic bone loss detection, gingival inflammation assessment, risk prediction, and monitoring); (vii) reported performance metrics (accuracy, sensitivity, specificity, AUC, F1, and correlation coefficients); (viii) presence or absence of external validation; (ix) reported limitations; and (x) ethical, legal, or regulatory considerations. For regulatory and grey literature sources, the following items were charted: issuing body; jurisdiction; year of adoption or entry into force; risk classification approach; transparency, human oversight, and post-market surveillance requirements; and applicability to AI-enabled medical devices in dentistry. The complete charted dataset is available from the corresponding author upon reasonable request.

### 2.9. Critical Appraisal of Individual Sources of Evidence

Consistent with PRISMA-ScR guidance [29], formal risk-of-bias appraisal of individual sources is not mandatory in scoping reviews; however, to strengthen the trustworthiness of conclusions about diagnostic performance evidence, the methodological quality of included diagnostic accuracy studies was appraised against the QUADAS-2 tool, and AI-specific reporting completeness was assessed against the APPRAISE-AI, MI-CLAIM, CONSORT-AI, and SPIRIT-AI checklists where applicable. For each applicable tool and study, an evidence-grounded draft judgement was first prepared by locating and reading the published source and applying the relevant tool’s criteria directly to what it reports, with AI assistance used for literature retrieval and first-pass drafting (disclosed in the Declaration of Generative AI and AI-assisted Technologies below); these draft judgements are then independently reviewed, confirmed, or amended by two reviewers (A.C., A.A.), with disagreements resolved by consensus, before the appraisal is treated as final. Findings of the appraisal informed the narrative synthesis, in particular the discussion of external validation limitations (Section 3.5) and methodological standardisation gaps (Section 4.1), but were not used to exclude sources from the review. The specific appraisal tool(s) applicable to each of the 29 included peer-reviewed studies, and the rationale where a given tool did not apply (for example, to evidence synthesis or non-AI sources), are mapped in Supplementary Table S6. No included primary study was a randomised trial of an AI intervention or an AI-specific trial protocol; CONSORT-AI and SPIRIT-AI were therefore not triggered by any individual source in the evidence base and are listed here as checklists that were considered but not applicable to the present set of included studies.

### 2.10. Synthesis of Results

Given the methodological, clinical, and statistical heterogeneity of the included sources, and consistent with the scoping nature of the review, a framework-based thematic synthesis approach was employed in place of quantitative meta-analysis. Seven primary thematic clusters were identified: (1) image-based AI for periodontal diagnosis; (2) non-clinical predictive modelling and biomarker-based screening; (3) digital patient monitoring and IoT applications; (4) data quality and external validation barriers; (5) regulatory and governance frameworks; (6) Kazakhstan and Central Asia contextual analysis; and (7) resources and infrastructure for AI deployment in Kazakhstan. Within each cluster, study findings were tabulated and summarised narratively, with attention to convergent and divergent patterns. To make the empirical evidence base directly assessable, the included empirical studies were charted into a structured summary reporting author/year, country, data type, AI method, sample size, validation type, and key reported performance; representative studies spanning all application domains are presented in Table 1, and the complete charting of the peer-reviewed empirical and review studies is provided in Supplementary Table S4. Reported performance metrics were presented as ranges (minimum–maximum) across studies rather than pooled, in recognition of the methodological heterogeneity documented in the underlying evidence base. No subgroup, sensitivity, or meta-regression analyses were performed; no statistical pooling was attempted. No assessment of reporting bias (e.g., funnel plot analysis) was applicable given the absence of quantitative synthesis. The thematic structure formed the basis for Section 3 (Results) and Section 4 (Discussion) of this manuscript. To avoid conflating heterogeneous evidence types in a single interpretive denominator, three tiers are distinguished throughout the synthesis and in Section 4: (i) the evidence map, comprising all 40 included sources; (ii) clinical performance evidence, restricted to 12 formally appraisable primary studies (11 AI/ML studies and one non-AI biomarker diagnostic accuracy study) for which QUADAS-2, APPRAISE-AI, and/or MI-CLAIM was judged applicable in Supplementary Table S6; and (iii) contextual/governance evidence, comprising evidence synthesis sources, regulatory and policy documents, patents, methodological guidance, and population context studies. Conclusions about diagnostic or predictive performance and clinical readiness are drawn only from tier (ii); tiers (i) and (iii) inform the narrative mapping and governance analysis but are not used as clinical performance evidence.

### 2.11. Certainty Assessment and Reporting Standards

Formal certainty-of-evidence grading (e.g., GRADE) was not undertaken because the synthesis is descriptive rather than quantitative, in line with PRISMA-ScR guidance [29]. Instead, the strength of evidence supporting each thematic conclusion is conveyed narratively, with explicit reference to study design, dataset size, presence or absence of external validation, and consistency across sources. Reporting of this review conforms to the PRISMA 2020 statement [28] and the PRISMA-ScR extension [29]; the completed PRISMA 2020 [30] and PRISMA-ScR checklists, with item-by-item indication of location within the manuscript, are provided in Supplementary Materials S1 and S2, respectively. The review was self-funded; no external sponsor had any role in the design, conduct, analysis, or reporting of this review. No competing interests are declared.

## 3. Results

### 3.1. Study Selection

The database search identified 54,134 records: PubMed (*n* = 10,189), Embase (*n* = 6694), Web of Science (*n* = 8412), Cochrane (*n* = 5457), and Scopus (*n* = 23,382). After removal of 37,898 duplicate records, 16,236 records underwent title and abstract screening, of which 16,192 were excluded, leaving 44 reports sought for retrieval as of the original search closure (30 April 2025); all 44 were retrieved and assessed for eligibility, of which 21 were excluded due to insufficient AI methodology description (*n* = 5), absence of periodontal-specific outcomes (*n* = 8), or purely opinion-based content without primary data (*n* = 8), leaving 23 sources included from the databases-and-registers stream as of the original search closure. In parallel, 16 records were identified through other methods within the original search window (eight grey literature and regulatory sources; eight WIPO PatentScope records, none of which had yet been granted/published as of 30 April 2025); of these, nine were excluded (eight patent applications not yet meeting eligibility as granted documents at that time; one cited as background only), leaving seven regulatory sources included from this stream. Together, the original search (closed 30 April 2025) yielded 30 included sources (23 from databases and registers plus seven from other methods). Ten further sources, all published after the 30 April 2025 search closure, were subsequently identified through expert peer review, citation checking, and reviewer requests during revision (Section 2.2), giving a final total of 40 information sources included in the qualitative synthesis (Figure 1): 29 peer-reviewed empirical and review studies and 11 regulatory, grey literature, and patent documents. Of the ten sources added during revision, six are peer-reviewed empirical/review/interpretive studies—Jafer et al. (2025) [9], Tan et al. (2025) [15] and its companion commentary Wu & Tsoi (2025) [16] (both on the PerioAI multimodal IOS–CBCT system), Yan et al. (2025) [31], Issilbayeva et al. (2025) [32], and Sarakbi et al. (2025) [33]—that were incorporated into the formal qualitative synthesis on the same eligibility basis as the sources identified by the original search (screened against the same criteria and charted in Supplementary Table S4); the other four are regulatory/grey literature/patent documents—Kazakhstan’s enacted AI Law [36], the national AI supercomputer infrastructure source [37], and two granted Russian patents, RU2841194 [34] (granted 3 June 2025) and RU2850170 [35] (granted 5 November 2025)—that were added specifically to update the regulatory and Kazakhstan context analysis (Section 3.6 and Section 3.8) and do not contribute to the diagnostic performance evidence base; the two patents had been provisionally located as pending applications during the original patent search but are reported here, on verification of their grant dates, as revision additions rather than original search inclusions. All ten nonetheless meet the same eligibility criteria as the sources identified by the original search and are reported, consistent with PRISMA 2020 guidance, as records identified by other methods in Figure 1 and itemised with exact publication dates in Supplementary Material S5. This revision update was a targeted, citation-triggered amendment rather than a full re-execution of the database and patent searches to a single new closure date (Section 2.2); the 54,134/16,236/44-record screening figures reported above describe the original search only and have not been regenerated for a later date, and this scope limitation is stated explicitly in Section 4.4. The original evidence set should accordingly be read as closed on 30 April 2025 (30 sources: 23 databases-and-registers + seven other methods); the ten later empirical, review, regulatory, and patent sources constitute a targeted supplementary update and do not themselves establish systematic search completeness beyond that date. The combined total of 40 literature sources is reported as a mapped evidence corpus, not as the product of a single continuous systematic search. The complete structured charting of the 29 peer-reviewed empirical and review studies is provided in Supplementary Table S4, and the 11 regulatory and governance documents are characterised in Table 2. The numbered reference list contains 50 entries because, in addition to these 40 included information sources, ten background and methodological works (for example, the WHO Global Oral Health Status Report and the PRISMA 2020 and PRISMA-ScR statements) are also cited; in a small number of instances closely related regulatory and policy materials were grouped under consolidated entries where appropriate.

A structured summary of representative studies from the empirical evidence base is presented in Table 1, charting each study by author/year, country, input data type, AI method, sample size, validation type, and key reported performance; the complete structured charting of the peer-reviewed empirical and review studies is provided in Supplementary Table S4. This summary makes the heterogeneity of model types, data modalities, and validation practices directly visible and supports the barrier analysis presented in Section 3.5.

### 3.2. Theme 1: Image-Based Deep Learning for Periodontal Diagnosis

#### 3.2.1. Radiographic Bone Loss Detection

Radiographic assessment of alveolar bone loss is the cornerstone of periodontitis staging under the 2018 AAP/EFP classification system, making it the most clinically impactful domain for AI application. An early CNN study by Krois et al. (2019) established that deep feed-forward CNNs trained on 2001 panoramic image segments achieved classification accuracy of 0.81, matching the mean performance of six experienced dentists [11]. Subsequent studies have substantially advanced both architectural complexity and performance.

Dujic et al. (2023) evaluated multiple Vision Transformer (ViT) architectures on periapical radiographs, reporting AUC values of 0.899–0.918 overall, with the highest performance for lower anterior teeth (AUC 0.944–0.970), demonstrating that transformer-based attention mechanisms provide contextual advantages over standard CNNs for localised bone level assessment [12]. Kurt-Bayrakdar et al. (2024) extended analysis to pattern-level detection on panoramic radiographs, identifying horizontal and vertical bone loss patterns and furcation involvement using a bespoke two-stage object detection architecture, achieving high sensitivity for Stage III/IV disease [44]. Xue et al. (2024) developed a DL pipeline for automated periodontitis staging on panoramic radiographs that integrated tooth segmentation with bone level quantification, achieving high diagnostic concordance with specialist assessment [45]. A conceptually distinct advance moves beyond two-dimensional radiographs altogether: Tan et al. (2025) demonstrated with PerioAI that fusing IOS surface meshes with CBCT volumes permits the gingiva–bone distance to be measured digitally and three-dimensionally, reproducing the information a clinician obtains by combining a periodontal probe with imaging but without physical probing and with sub-millimetre precision (0.040 mm error) across 2507 patients [15]. This positions multimodal IOS–CBCT analysis not merely as an adjunct to radiographic interpretation but as a promising candidate complement to, and potential future adjunct or partial alternative for, manual probing, which captures three-dimensional information on attachment and bone loss that single-modality two-dimensional models cannot, pending independent replication and clinical validation beyond the single multicentre study reported to date [16]. Its principal translational constraint is its dependence on paired CBCT and IOS acquisition, which carries cost, radiation dose, and equipment availability implications that are especially salient for resource-constrained settings.

The systematic review and meta-analysis by Khubrani et al. (2025)—the most methodologically rigorous synthesis to date, employing APPRAISE-AI quality assessment—analysed studies from 1990 to January 2024 across five databases [21]. Pooled sensitivity was high across CNN-based models, but marked heterogeneity in study design, training dataset characteristics, and reporting standards was identified. The review specifically noted that few studies reported external validation data, limiting conclusions about real-world generalisability.

#### 3.2.2. Gingival Inflammation Assessment from Intraoral Images

Visual assessment of gingival inflammation—historically dependent on clinician judgment using indices such as the Gingival Index and Bleeding on Probing—represents a second major application domain. Li et al. (2025) developed GC-U-Net, which combined IOS data with a global context attention module to enable automated segmentation and scoring of gingival inflammation, achieving strong correlation with clinical bleeding indices [14]. The Faster R-CNN model by Alalharith et al. (2020) applied to orthodontic patients achieved 77.12% accuracy for gingivitis detection, establishing DL viability for preventive monitoring in high-risk subpopulations [17].

Multi-disease AI platforms integrating periodontal with broader oral pathology assessment have also emerged. Kim et al. (2022) deployed a Korean web-based system using Faster R-CNN with Inception and ResNet backbones to simultaneously diagnose five oral disease types from panoramic radiographs in real clinical workflow, representing one of the most advanced examples of clinically integrated AI diagnostic support in dentistry [13].

### 3.3. Theme 2: Machine Learning for Non-Clinical Predictive Screening

A clinically distinct but complementary paradigm employs ML algorithms on non-imaging data for determinant-based, population-level periodontal risk assessment and screening—addressing settings where radiographic equipment is unavailable or where pre-referral triage is needed.

Deng et al. (2024) developed a multiclass ML screening tool in Shanghai and Hong Kong that combined patient-reported parameters (gingival condition, tooth mobility, and bleeding on brushing) with salivary MMP-8 and haemoglobin biomarkers from a simple oral rinse test [19]. Using random forest and gradient boosting algorithms, the model discriminated between periodontal health, gingivitis, Stage I/II periodontitis, and Stage III/IV periodontitis with clinically meaningful accuracy, demonstrating a viable pathway for non-invasive community screening. Deng et al. (2022) further validated the MMP-8 oral rinse test as a point-of-care biomarker, confirming its capacity to reflect current disease activity rather than historical tissue destruction, which is a critical advantage over conventional probing [39].

Bashir et al. (2022) performed a rigorous comparative ML study, evaluating ten algorithms across U.S. National Health and Nutrition Examination Survey (NHANES) and Taiwanese national datasets [18]. The study demonstrated that model performance consistently degraded during cross-national external validation despite high internal accuracy—a finding with profound implications: current ML predictive models for periodontitis cannot be safely deployed in new populations without local revalidation. The study also identified that commonly used predictors (sociodemographic variables, self-reported oral health status) are too coarse for complex algorithms, and that datasets of hundreds of thousands to millions of records are required for models to fully leverage ML capabilities.

The association between periodontal inflammation and systemic health indicators has been further explored using ML approaches; Yan et al. (2025) applied ML to quantify the relationship between periodontal inflamed surface area (PISA) and systemic biomarkers including C-reactive protein, demonstrating the potential for AI-mediated integration of periodontal and systemic health monitoring [31].

### 3.4. Theme 3: Digital Patient Monitoring and IoT Applications

Beyond clinical diagnosis, AI is being integrated into continuous patient management platforms that operate between dental visits to address the persistent challenge of patient adherence, which is a major determinant of periodontal treatment outcomes.

Tonetti et al. (2020) reported in a supportive periodontal care population that self-reported bleeding on brushing captured by intelligent power-driven toothbrushes connected to a mobile application was associated with bleeding on probing at the next clinical visit [20]. The analysis used generalised mixed-effects and stepwise regression rather than a trained machine learning model. Accordingly, this study is retained as contextual evidence for the feasibility of IoT-based home monitoring between recall appointments, but it is not included in the AI/ML clinical performance appraisal tier.

AI-powered chatbot systems have emerged as another patient-engagement tool. Patents from the Russian Federation (RU2841194; RU0002850170) describe systems providing personalised oral hygiene reminders via chatbot interfaces, where clinician-recorded videos and individualised instructions are delivered through unique patient identifiers—enabling a shift from episodic consultation to continuous, personalised support [34,35]. Teledentistry platforms further extend digital monitoring to remote and underserved populations: Kengne Talla et al. (2024) conducted a systematic review protocol confirming teledentistry’s evidence-supported potential for improving both access and quality of oral health care, particularly in geographically dispersed populations [42].

The ‘virtual patient’ concept—integrating data from IOS, 3D facial scanning, CBCT, and IoT devices into a unified digital twin—represents the frontier of this monitoring paradigm [50]. However, Shuto et al. (2025) noted in a scoping review that standardised protocols for integrating diverse digital dental records remain absent, and that this gap will increasingly constrain AI-powered comprehensive treatment planning [38].

### 3.5. Theme 4: Data Quality, Standardisation, and External Validation Barriers

A consistent finding across all reviewed studies was the fundamental constraint of data quality and dataset limitations on AI model reliability and generalisability. Four interconnected barriers were identified:

First, dataset scale insufficiency: The majority of DL studies in periodontology used fewer than 5000 images for training, which is far below the scale needed to train robust, generalisable models. Bashir et al. (2022) explicitly calculated that hundreds of thousands to millions of records are required for complex ML algorithms to reach their performance potential [18].

Second, absence of external validation: The systematic review by Ferrara et al. (2025) found that most included DL studies validated models only on internal test sets from the same institution or country, with no external validation on independent populations [7]. This renders accuracy metrics poorly predictive of real-world performance.

Third, data heterogeneity and labelling inconsistency: The lack of standardised radiographic acquisition protocols, image quality standards, and annotation conventions across institutions produces training data variability that degrades model performance. Gusev et al. (2022) articulated standardised methodological requirements for ML model development and validation reporting—including MI-CLAIM-compliant documentation of training datasets, validation procedures, and performance metrics—as a prerequisite for clinical-grade model transparency [40].

Fourth, LMIC data underrepresentation: Umer et al. (2024) confirmed the near-total absence of AI dentistry research from low-middle income countries, meaning that models trained predominantly on data from East Asian, European, and North American populations are poorly validated for morphological, behavioural, and epidemiological characteristics of these populations [22]. This represents both a scientific gap and an equity concern.

### 3.6. Theme 5: Regulatory and Governance Frameworks

Table 2 presents a comparative synthesis of key international and national AI regulatory frameworks relevant to periodontal clinical deployment.

The EU AI Act, enacted on 12 July 2024, establishes the most stringent legally binding framework, classifying medical AI diagnostic systems—including periodontal imaging AI—as high-risk and requiring mandatory conformity assessment, comprehensive technical documentation, algorithmic logging for traceability, and continuous post-market monitoring plans [23]. The U.S. FDA has developed a parallel risk-based system through the SaMD pathway, with the 2023 introduction of the Predetermined Change Control Plan (PCCP) specifically addressing the challenge of adaptive AI systems that modify their behaviour through post-deployment learning [24].

Kazakhstan’s regulatory trajectory offers a notable case for analysis. The AI Development Concept for 2024–2029, approved by Government Resolution No. 592 of July 24, 2024, establishes the creation of national AI databases, development of a dedicated AI law, and alignment with international governance principles as strategic priorities [25]. The dedicated statute has since been enacted: Law No. 230-VIII ‘On Artificial Intelligence’, signed on 17 November 2025 and in force from 18 January 2026, introduces a binding risk-based classification, lifecycle risk management, transparency obligations, an audited registry of trusted high-risk systems, and liability and insurance provisions for AI-related harm [36]. At the operational level, Order of the Minister of Healthcare No. KP ДCM-39 (2021) defines requirements for electronic information resources in telemedicine services, while Order No. 199/HҚ of the Ministry of Digital Development (2023) operationalises personal data processing rules for operators [48,49]. The intersection of these instruments with now-enacted AI-specific legislation creates a regulatory opportunity to establish Kazakhstan-specific validation and certification pathways for dental AI tools.

### 3.7. Theme 6: Kazakhstan and Central Asia—Contextual Analysis

To preserve interpretive clarity, this section distinguishes findings directly supported by local Kazakhstani evidence from those inferred by analogy from international frameworks. Statements grounded in Kazakhstan-specific primary or regulatory evidence are explicitly attributed to national sources [25,32,48,49]; statements that extrapolate from international frameworks (EU AI Act, FDA SaMD, WHO, and UNESCO) are identified as inferential and are not presented as locally validated. No peer-reviewed clinical study evaluating an AI periodontal tool in a Kazakhstani population was identified; accordingly, all clinical performance expectations for Kazakhstan are inferential pending local validation.

Drawing on local evidence, Kazakhstan’s healthcare system context is characterised by several factors with direct relevance to AI integration in periodontology. The country’s healthcare system is in active digital transformation under the eHealth programme. A high caries burden and a limited specialist periodontal workforce outside the major urban centres (Astana, Almaty, and Shymkent) are features commonly reported for upper-middle-income transitional economies; we note, however, that no national periodontal epidemiological survey for Kazakhstan meeting our eligibility criteria was identified, and this evidentiary gap is itself treated as a constraint on Stage 1 of the proposed framework. A recent Kazakhstani study characterising oral microbiome patterns associated with dental caries in adolescents provides local biological context for preventive applications; it was not designed to quantify unmet oral health need, and is not cited here as evidence of service-level need [32].

The digital infrastructure available in Kazakhstan has expanded significantly; telemedicine frameworks are legally established, national health information systems are in development, and the Digital Kazakhstan programme has invested in telecommunications connectivity across oblasts. Elaborating on the significance of this expansion for oral health specifically: the national eHealth architecture provides the structured electronic health record (EHR) backbone into which AI diagnostic outputs and periodontal charting can be embedded, enabling longitudinal capture of clinical attachment loss, probing depth, and radiographic bone-level data at the population scale required for risk modelling. Legally established telemedicine and teledentistry frameworks [48] extend specialist periodontal triage to rural oblasts where periodontal workforce density is lowest, allowing AI-assisted pre-referral screening to function as a force multiplier for a constrained specialist workforce. Investment in cross-oblast connectivity makes IoT-based home monitoring (e.g., connected toothbrushes) and chatbot-mediated adherence support technically feasible at distance, supporting the shift from episodic to continuous periodontal care. Critically, a unified national EHR also constitutes the de-identified data substrate from which a representative Kazakhstani periodontal dataset can be assembled, which is the foundational precondition (Stage 1) for locally valid AI. In this sense, the digital infrastructure expansion is not merely a delivery channel but the enabling platform for population-level periodontal risk surveillance and the data sovereignty prioritised by the AI Development Concept [25].

However, as confirmed by the AI-in-LMIC literature [22], critical gaps persist in structured clinical dental data repositories, radiographic imaging standardisation across facilities, and training of dental professionals in digital tools and AI output interpretation. These constraints are inferred to apply to Kazakhstan by analogy with comparable transitional economies and have not yet been quantified in published Kazakhstani primary studies.

Within the Central Asia and post-Soviet regional context, Kazakhstan has among the more developed AI regulatory frameworks in the region, making it a potential reference point for Kyrgyzstan, Uzbekistan, Tajikistan, and Azerbaijan, though a systematic regional benchmarking exercise has not been undertaken here. The involvement of co-authors from the International Higher School of Medicine (Bishkek, Kyrgyzstan) and Osh State University in the present study reflects this regional collaborative dimension and suggests pathways for multinational data sharing to address the dataset scale limitations identified in the evidence base.

### 3.8. Resources and Infrastructure for AI Deployment in Kazakhstan: Chances and Risks

Translating the global evidence into a Kazakhstani periodontal AI programme depends on the national resource and infrastructure base now in place. Since 2024, Kazakhstan has assembled the principal building blocks of an AI ecosystem: a national supercomputing capability, the Alem.cloud cluster in Astana, launched in July 2025 and built on NVIDIA H200 GPUs (HPE Cray XD670 servers) within a Tier III data centre and accessible to universities, start-ups, and research centres [37], complemented by an academic supercomputer network across leading universities; a National AI Platform offering shared data libraries, model training tools, and controlled computer access; sovereign Kazakh-language large language models (KazLLM and the Sherkala family); and a dedicated Ministry of Artificial Intelligence and Digital Development established in 2025 to coordinate the sector [25]. Connectivity and digital government penetration are high—internet access approaches that of leading economies and the great majority of public services are delivered electronically—providing the population reach required for teledentistry, IoT-based home monitoring, and population-level surveillance. For periodontology specifically, the national eHealth electronic health record architecture supplies the structured backbone into which de-identified intraoral scan, panoramic radiograph, CBCT, and charting data could be aggregated to assemble the representative national dataset that Stage 1 of the proposed framework requires.

The regulatory base has also matured beyond the 2024–2029 AI Development Concept [25]. On 17 November 2025, Kazakhstan enacted Law No. 230-VIII ‘On Artificial Intelligence’ (in force from 18 January 2026), one of the first comprehensive national AI statutes worldwide. The Law adopts a risk-based classification in which obligations intensify for high-risk systems, including those affecting human health; mandates lifecycle risk management, technical documentation, transparency, and labelling of synthetic outputs; introduces an application-based ‘trusted’ high-risk registry requiring independent audit; and addresses liability and insurance for harm caused by AI systems—the very governance elements that the implementation framework (Section 4.2, Stages 2 and 6) identifies as prerequisites [36]. Medical diagnostic AI for periodontology would foreseeably fall within the high-risk tier, aligning national requirements with the EU AI Act and FDA SaMD logic analysed above [23,24].

Against these advantages stand material constraints. Domestic high-performance computing resources, although now substantial, remain dependent on imported GPUs and strategic partnerships, limiting the capacity to train large multimodal models at scale domestically. The data substrate is immature: structured clinical dental repositories, harmonised radiographic acquisition standards, and annotation conventions are largely absent, and no Kazakhstani periodontal imaging dataset has been published—a gap inferred by analogy with comparable transitional economies [22]. Workforce readiness is limited, with few dental professionals trained in AI output interpretation, uncertainty handling, and override. Cybersecurity exposure is rising, and personal health data processing must comply with sectoral data protection rules [48]. Finally, resource-intensive modalities such as the paired CBCT and IOS acquisition required by systems like PerioAI [15,16] are concentrated in major urban centres, risking an urban–rural equity gap unless deployment is sequenced with teledentistry-based triage. Table 3 summarises these resource and infrastructure dimensions as paired chances and risks.

In sum, Kazakhstan has assembled several enabling elements relevant to transitional economies—sovereign compute, a national data and AI platform, a high-reach digital health system, and now binding AI legislation—while facing data maturity, workforce, equity, and security constraints that the six-stage framework is explicitly designed to sequence and mitigate.

## 4. Discussion

### 4.1. The Dual Reality: Technological Promise vs. Implementation Prerequisites

The synthesised evidence presents a dual reality. On the technological side, AI—particularly DL for radiographic image analysis and ML for non-clinical predictive modelling—has achieved proof-of-concept across multiple periodontal application domains, with CNN-based models matching or approaching specialist diagnostic accuracy in controlled settings. The methodological diversity spanning Faster R-CNN, GC-U-Net, Vision Transformers, random forests, and gradient boosting algorithms reflects the maturity of the research field. On the implementation side, however, AI in periodontology remains pre-clinical at scale: few systems have been validated externally, fewer still are CE/FDA-cleared, and real-world clinical deployment outside controlled pilot environments is rare.

This implementation gap is not primarily a technological deficiency: it is a governance deficiency. As foreshadowed in Section 1.4, the absence of operationalised regulatory frameworks in transitional economies has concrete downstream effects: it leaves liability for algorithmic error unallocated between developers, institutions, and clinicians; it discourages clinical adoption by removing certification pathways; and it constrains insurance coverage for AI-assisted care. The evidence demonstrates that the path to clinical deployment requires: (1) vastly larger and more representative training datasets incorporating diverse ethnic, geographic, and socioeconomic populations; (2) standardised data collection, annotation, and reporting protocols aligned with MI-CLAIM, CONSORT-AI, and SPIRIT-AI; (3) rigorous external and cross-national validation as a precondition—not an afterthought—for clinical deployment; (4) regulatory certification under applicable national frameworks; and (5) a legal infrastructure that assigns liability, mandates insurance, and protects patient rights in AI-assisted clinical encounters. Throughout this discussion, statements about diagnostic or predictive performance draw only on the clinical performance evidence tier defined in Section 2.10—the 12 formally appraisable primary studies (11 AI/ML studies and one non-AI biomarker diagnostic accuracy study) for which QUADAS-2, APPRAISE-AI, and/or MI-CLAIM was judged applicable (Section 2.9). Regulatory, patent, review, and population context sources inform governance analysis but are not treated as clinical performance evidence.

### 4.2. The Proposed Six-Stage Implementation Framework

**Figure 2:**
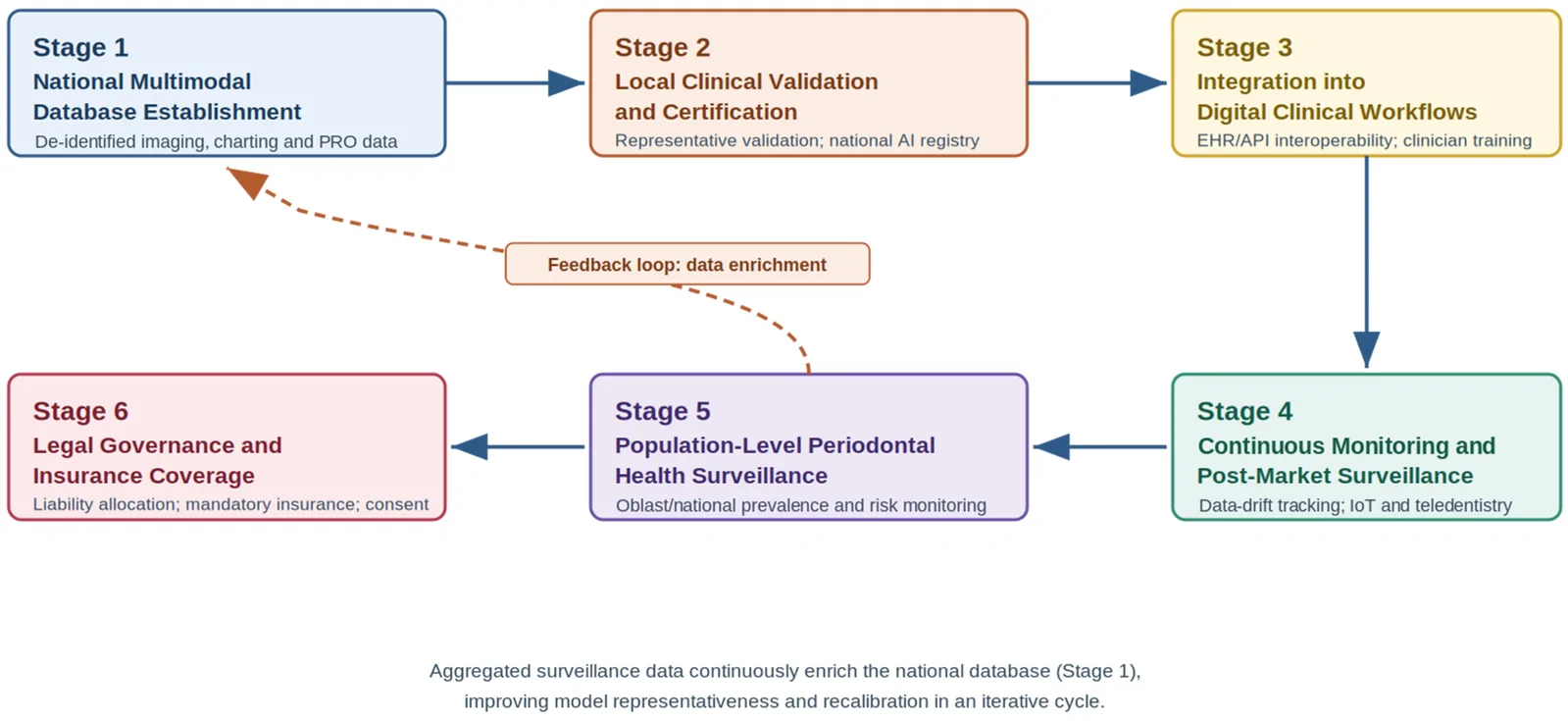
Process flowchart of the proposed six-stage implementation framework. The framework comprises six sequential stages: National Multimodal Database Establishment (Stage 1); Local Clinical Validation and Certification (Stage 2); Integration into Digital Clinical Workflows (Stage 3); Continuous Patient Monitoring and Post-Market Surveillance (Stage 4); Population-Level Periodontal Health Surveillance (Stage 5); and Legal Governance and Insurance Coverage (Stage 6). The dashed feedback arrow from Stage 5 to Stage 1 denotes the iterative data enrichment loop, whereby aggregated population surveillance data continuously enrich the national database to improve model representativeness and recalibration.

Synthesising the technical evidence, governance requirements, and Kazakhstan’s contextual characteristics, we propose a six-stage evidence-based implementation framework (Figure 2). A dedicated population-level periodontal health surveillance stage (Stage 5) is incorporated because the determinant-based risk assessment evidence (Section 1.2 and Section 3.3) and the governance and prevention objectives of this review require an explicit community surveillance function that is distinct from individual-patient post-market monitoring. Each stage is grounded in specific literature findings and regulatory requirements:

Stage 1—National Multimodal Database Establishment: The consistent finding that AI model performance degrades sharply during external validation [18] mandates the creation of large-scale national clinical datasets as a foundational precondition. In the Kazakhstani context, this involves the collection of de-identified multimodal data—intraoral scans, panoramic radiographs, CBCT, clinical charting data, and patient-reported outcomes—from medical institutions across oblasts, with harmonisation to a unified quality standard. This aligns with the AI Development Concept’s priority of data infrastructure and technological sovereignty [25]. Personal data collection must strictly comply with Order No. 199/HҚ of the Ministry of Digital Development [48], with cryptographic protection, access auditing, and anonymisation protocols implemented from the outset.

Stage 2—Local Clinical Validation and Certification: AI systems must undergo mandatory clinical validation on a population representative of Kazakhstan before deployment, following the methodology standards outlined by Gusev et al. (2022) for ML model scientific reporting [40]. Risk classification of AI products should align with the EU AI Act’s high-risk category for medical diagnostic AI [23], with inclusion in a Unified National Registry of Medical AI Solutions providing regulatory clarity for developers and clinicians alike. This stage addresses the critical absence of locally validated models that characterises the current global evidence base.

Stage 3—Integration into Digital Clinical Workflows: Certified AI systems should be integrated into existing electronic health record systems of hospitals and private dental practices, with standardised application programming interfaces (APIs) ensuring interoperability. This requires the development of unified protocols for integrating data from IOS, 3D facial scanning, radiographic systems, and IoT monitoring devices—addressing the gap in such standards identified by Shuto et al. (2025) [38]. Digital workflow integration should be accompanied by structured training of dental professionals in AI output interpretation, uncertainty quantification, and override protocols.

Stage 4—Continuous Patient Monitoring and Post-Market Surveillance: Following clinical integration, systems must be enrolled in continuous post-market surveillance programs that track real-world performance, identify data drift (degradation of model accuracy over time as clinical populations evolve), and monitor for algorithmic errors. IoT-based patient monitoring platforms—including intelligent toothbrush systems [20] and chatbot-mediated adherence support [34]—should be deployed as components of periodontal maintenance protocols, where AI has been shown to support precision monitoring and personalised supportive care [33], shifting care from episodic clinic encounters to continuous patient engagement. Teledentistry platforms [42] should be leveraged to extend monitoring coverage to rural oblasts; however, professional-, organisational-, and infrastructure-level barriers to teledentistry implementation must be anticipated and addressed during roll-out [41].

Stage 5—Population-Level Periodontal Health Surveillance: Building on the individual-level monitoring of Stage 4, this stage establishes a community/population dental-health surveillance function dedicated to periodontal disease. Aggregating de-identified, determinant-based risk indicators (Section 1.2)—clinical charting, patient-reported behaviour, salivary biomarkers, and AI-derived radiographic findings—across the national EHR enables continuous estimation of periodontal disease prevalence, incidence, and risk distribution at oblast and national levels. This supports three governance and prevention objectives directly: (i) it provides the epidemiological feedback loop needed to target preventive resources to high-risk communities, including the rural and adolescent populations with documented unmet need [32]; (ii) it operationalises the population-level risk assessment objective of this review and the WHO and UNESCO emphases on equitable, accountable, population-beneficial AI [46,47]; and (iii) the surveillance dataset itself enriches the national multimodal database (Stage 1), creating a virtuous cycle in which population monitoring continuously improves model representativeness and recalibration. Surveillance must operate under the same data sovereignty, anonymisation, and access-audit safeguards mandated in Stage 1 [48].

Stage 6—Legal Governance and Insurance Coverage: The final and essential stage addresses the legal infrastructure without which clinical AI adoption remains precarious. Kazakhstan’s enacted AI Law (No. 230-VIII, in force from 18 January 2026), which aligns with EU AI Act principles [25,36], and its implementing rules and sectoral medical device pathways must explicitly address: liability allocation between AI developers, medical organisations, and individual clinicians for algorithmic errors; mandatory professional liability insurance extending to AI-assisted clinical decisions; and informed consent requirements ensuring patients understand when algorithmic tools are used in their care. The formalisation of these provisions—drawing on WHO guidance [46] and UNESCO principles [47]—creates the predictable legal environment necessary for sustainable AI ecosystem development.

### 4.3. Unique Contribution: A Proposed Conceptual Implementation Framework for Transitional Economies

The contribution of the present review that distinguishes it from the existing literature is its explicit focus on governance and implementation feasibility in a transitional economy context. To state the take-home message plainly: the primary aim of this work is not to re-benchmark AI diagnostic performance but to propose an operationalised utilisation and governance structure through which validated AI can be safely deployed in Kazakhstan, with the global evidence synthesis serving to justify and calibrate that structure. To the best of our knowledge, while prior reviews have systematically catalogued AI performance metrics in high-income country settings, none has proposed an operationalised, contextually grounded implementation pathway for an emerging economy actively developing its regulatory infrastructure. Kazakhstan’s combination of characteristics—simultaneously experiencing rapid healthcare digitalisation, developing AI-specific legislation, an active commitment to international regulatory alignment, and the clinical workforce and infrastructure constraints characteristic of middle-income and transitional economies—makes it a potentially transferable case for developing and evaluating such a framework, with lessons that may be relevant to other emerging economies pursuing comparable digital health and AI governance transitions.

The framework is designed to be iteratively adaptable: each stage generates evidence (national validation data, certified product registries, monitoring datasets, and litigation precedents) that feeds back to improve subsequent stages. This feedback architecture mirrors the Total Product Lifecycle approach mandated by the FDA [24] and the continuous monitoring requirements of the EU AI Act [23], ensuring that the framework evolves with both the evidence base and the regulatory environment.

### 4.4. Limitations

Several limitations of this review should be acknowledged. First, the absence of published peer-reviewed clinical studies from Kazakhstan specifically evaluating AI periodontal tools means that the Kazakhstan-specific analysis is based on regulatory and epidemiological evidence rather than primary clinical data. This distinction is made explicit in Section 3.7 and supports the research priority this review aims to highlight. Second, the rapid evolution of both AI technology and regulatory frameworks (particularly the EU AI Act, which began phased implementation in 2024) means that some specific provisions cited may be subject to further elaboration through implementing regulations. Third, language restrictions (English and Russian) may have introduced selection bias, potentially missing relevant studies published in other languages from Central Asia contexts. Fourth, the post-30-April-2025 update that incorporated ten additional sources (Section 2.2 and Section 3.1) was a targeted, citation- and peer-review-triggered amendment rather than a full re-execution of the five-database and patent search to a single later closure date; formal search completeness therefore cannot be claimed beyond 30 April 2025 for the original streams, and the true universe of eligible post-cutoff literature may be larger than the ten sources identified here. Fifth, as a systematic scoping review, this work maps and synthesises evidence narratively and does not provide pooled effect estimates; readers seeking quantitative diagnostic accuracy meta-estimates are referred to the dedicated meta-analyses cited herein [7,21].

## 5. Conclusions

This systematic scoping review synthesised evidence across 40 sources to address the critical gap between AI’s demonstrated proof-of-concept in periodontology and its safe, ethical, and clinically effective deployment in real-world practice, with particular focus on Kazakhstan and analogous transitional economies.

The evidence indicates that AI tools—particularly CNNs and Vision Transformers for radiographic bone loss detection, ML models for non-clinical screening, and IoT platforms for continuous patient monitoring—represent a significant and rapidly maturing technological advance with the potential to expand the capacity for objective, scalable, preventive periodontal care. However, external validation performance consistently degrades relative to internal benchmarks, underscoring that accuracy figures from controlled studies cannot be extrapolated to clinical deployment without local validation on representative populations.

The regulatory convergence of the EU AI Act, U.S. FDA framework, WHO guidance, and Kazakhstan’s AI Development Concept 2024–2029 around risk-based classification, algorithmic transparency, and lifecycle surveillance shows broad convergence toward a common governance framework for AI medical systems. Kazakhstan’s recently enacted AI legislation (Law No. 230-VIII) and its active healthcare digitalisation program create a current policy window to translate these international principles into a nationally operationalised certification and monitoring system for dental AI tools.

The proposed six-stage implementation framework—progressing from national database infrastructure through local validation, digital workflow integration, continuous monitoring, population-level periodontal health surveillance, and robust legal governance—provides a proposed pathway for prospective evaluation and adaptation by Kazakhstan and other transitional economies. Future research priorities include: the creation of large-scale, nationally representative Kazakhstani periodontal clinical datasets; prospective multicentre clinical trials evaluating AI-assisted versus conventional periodontal diagnosis; development of standardised protocols for multimodal digital data integration; and legal scholarship clarifying liability and insurance mechanisms for AI-assisted dental encounters.

In conclusion, the integration of artificial intelligence into periodontology is not a distant aspiration: it is a realistic and worthwhile medium-term priority that requires coordinated investment in data infrastructure, rigorous science, principled governance, and legal innovation. Kazakhstan’s evolving regulatory and digital health infrastructure position it to contribute meaningfully to regional progress on this agenda, provided the local clinical validation identified as a limitation in this review (Section 4.4) is prioritised.

## Figures and Tables

**Table 1:** Summary of representative empirical and review studies of AI applied to periodontal diagnosis, risk prediction, and monitoring (author/year, country, data type, AI method, sample size, validation type, and key reported performance). The complete structured charting of the peer-reviewed empirical and review studies is provided in Supplementary Table S4. The companion commentary to the PerioAI study (Wu & Tsoi 2025 [16]), which interprets the IOS–CBCT digital-probing approach, is cited in the main text and is not charted as a separate empirical row.

Author (Year)	Country	Data Type	AI Method/Architecture	Sample Size	Validation Type	Key Reported Performance
Krois et al. (2019) [11]	Germany	Panoramic radiograph segments	Deep feed-forward CNN (7-layer)	2001 image segments	Internal (cross-validation)	Accuracy 0.81 for PBL; matched mean of 6 dentists (0.76)
Dujic et al. (2023) [12]	Germany	Periapical radiographs	Vision Transformer (ViT)	Periapical radiograph dataset	Internal	Accuracy 83.4–85.2%; AUC 0.899–0.918 (lower anterior AUC up to 0.970)
Kim et al. (2022) [13]	South Korea	Panoramic radiographs	Faster R-CNN + Inception + ResNet	Hospital panoramic dataset	Internal (clinical workflow)	Real-time multi-disease detection (5 oral pathologies); separate model per disease
Li et al. (2025) [14]	China	Intraoral scanning (IOS)	GC-U-Net (global context attention)	IOS gingival image dataset	Internal	r = 0.836 (Sulcus Bleeding Index); r = 0.618 (Bleeding Index)
Tan et al. (2025) [15]	China (multicentre)	Intraoral scanning (IOS) + CBCT	Full-stack DL (IOS + CBCT segmentation, multimodal fusion, digital probing)	2507 patients	Internal and external	Automated 3D gingiva–bone distance; digital probing error 0.040 mm; tooth-level volumetric diagnosis
Alalharith et al. (2020) [17]	Saudi Arabia	Intraoral photographs (orthodontic)	Faster R-CNN + ResNet-50	Orthodontic patient images	Internal	Accuracy 77.12% for early gingivitis detection
Kurt-Bayrakdar et al. (2024) [44]	Turkey	Panoramic radiographs	Two-stage object detection (DL)	Retrospective panoramic dataset	Internal	High sensitivity for horizontal/vertical bone loss patterns and furcation; Stage III/IV
Xue et al. (2024) [45]	China	Panoramic radiographs	DL pipeline (segmentation + bone-level quantification)	Panoramic dataset	Internal	High concordance with specialist staging
Bashir et al. (2022) [18]	USA/Taiwan	NHANES + Taiwanese national data	10 ML algorithms (comparative)	Nationally representative datasets	External (cross-national)	High internal accuracy; significant external validation performance drop
Deng et al. (2024) [19]	China (Shanghai/Hong Kong)	Patient-reported + salivary MMP-8/Hb	Random forest, gradient boosting (multiclass)	Community screening cohort	Internal	Discriminated health/gingivitis/Stage I–II/Stage III–IV
Deng et al. (2022) [39]	China	Saliva/oral rinse biomarkers	Point-of-care biomarker (aMMP-8) analysis	Diagnostic accuracy cohort	Internal	Reflected current disease activity vs historical destruction
Tonetti et al. (2020) [20]	Multinational (IoT network)	IoT toothbrush + patient-reported	IoT observational monitoring; mixed-effects/stepwise regression	Supportive periodontal care population	N/A (non-ML prospective observational study)	Self-reported bleeding on brushing predicted bleeding on probing
Yan et al. (2025) [31]	UK	Periodontal + systemic biomarkers	ML (association modelling)	Clinical/biomarker dataset	Internal	Quantified PISA–CRP and systemic-marker associations
Khubrani et al. (2025) [21]	Multinational (review)	2D dental radiographs (review)	Systematic review/meta-analysis (APPRAISE-AI)	Studies 1990–2024	N/A (evidence synthesis)	Pooled high sensitivity; marked heterogeneity; few external validations
Ferrara et al. (2025) [7]	Italy (review)	Radiographic DL (review)	Systematic review (QUADAS-2)	13 studies appraised	N/A (evidence synthesis)	Accuracy range 73.0–98.6%; most lacking external validation
Scott et al. (2023) [6]	UK (review)	Mixed AI in periodontology (review)	Scoping review	50 included studies	N/A (evidence synthesis)	Heterogeneous reporting outcomes across studies
Umer et al. (2024) [22]	Pakistan (LMIC review)	AI in dentistry, LMICs (review)	Scoping review	25 of 1587 records (LMICs)	N/A (evidence synthesis)	Severe underrepresentation of LMICs/transitional economies
Shuto et al. (2025) [38]	Japan (review)	Facial scans/digital records (review)	Scoping review	Facial scan literature	N/A (evidence synthesis)	Absence of standardised multimodal integration protocols

*Abbreviations*: AUC, area under the ROC curve; CBCT, cone beam computed tomography; CNN, convolutional neural network; CRP, C-reactive protein; DL, deep learning; IOS, intraoral scanning; IoT, Internet of Things; ML, machine learning; MMP-8, matrix metalloproteinase-8; NHANES, National Health and Nutrition Examination Survey; PBL, periodontal bone loss; PISA, periodontal inflamed surface area; ViT, Vision Transformer. N/A indicates an evidence synthesis (review) source for which primary validation is not applicable.

**Table 2:** Comparative analysis of international and national AI regulatory frameworks for healthcare.

Framework/Jurisdiction	Risk Classification Approach	Key Requirements	Transparency and Explainability	Post-Market Surveillance	Refs.
EU AI Act (2024/1689)	Risk-based (Prohibited/High-Risk/Limited/Minimal)	Conformity assessment; technical documentation; human oversight; CE marking for high-risk	Mandatory for high-risk AI; logs for traceability	Mandatory; continuous post-market monitoring plan required	[23]
U.S. FDA SaMD Framework	Risk-based (Class I/II/III) aligned with medical device classification	Pre-market submission (510(k), De Novo, PMA); Predetermined Change Control Plan (PCCP)	Good Machine Learning Practice (GMLP) principles; encouraged transparency	Total Product Lifecycle (TPLC) approach; ongoing real-world evidence collection	[24]
WHO AI Ethics Guidance (2021)	Principles-based (ethics and human rights foundation)	Fairness, inclusiveness, accountability, sustainability, governance of data	Central to trust-building; explainability as ethical imperative	Implied within responsible and accountable use principle	[46]
UNESCO Recommendation on AI Ethics (2021)	Proportionality and do-no-harm approach; multi-stakeholder governance	Safety, security, human rights protection; environmental sustainability	Core principle for public trust and beneficial outcomes	Linked to accountability and continuous assessment principles	[47]
Kazakhstan AI Development Concept 2024–2029 (Res. No. 592)	Enacted risk-based model (Law No. 230-VIII, 2025) aligning with international standards; national registry of trusted high-risk AI systems and registry of AI medical solutions envisaged	Data sovereignty; mandatory local clinical validation; digital sovereignty; binding AI Law in force from 18 January 2026 with lifecycle risk management, technical documentation, and audited trusted high-risk registry	Required for market access, clinical trust, and patient safety	Mandated under continuous lifecycle risk management; follows EU and international standards	[25,36,48,49]

**Table 3:** Resources and infrastructure for AI in periodontology in Kazakhstan: paired chances (advantages) and risks (disadvantages).

Resource/Infrastructure Dimension	Chance/Advantage	Risk/Disadvantage	Refs.
Computing (Alem.cloud national supercomputer; academic network; National AI Platform)	Sovereign capacity to train and host multimodal periodontal models and serve inference nationally.	Dependence on imported GPUs and external partnerships; finite domestic capacity to scale large-model training.	[25,37]
National data infrastructure (eHealth EHR; data libraries)	Backbone to aggregate de-identified IOS, panoramic, CBCT, and charting data for a representative national dataset (Stage 1).	Structured dental repositories, imaging standardisation, and annotation conventions largely absent; no published Kazakhstani periodontal dataset.	[22,25,48]
Connectivity and e-government reach	High internet penetration and electronic public services enable teledentistry, IoT home monitoring, and population surveillance at scale.	Urban–rural disparities in device access and specialist coverage may concentrate benefit in major cities.	[25,42]
Regulatory framework (AI Concept 2024–2029; Law No. 230-VIII on AI)	Risk-based statute with lifecycle risk management, transparency, a trusted high-risk registry, and liability/insurance provisions, aligning with EU/FDA logic.	Implementing rules, sectoral medical device pathways, and audit capacity are still being operationalised; compliance burden for developers.	[23,24,25,36]
Human capital and clinical readiness	National AI education expansion (university curricula, training centres) building a future workforce.	Few dental professionals currently trained in AI output interpretation, uncertainty handling, and override.	[25]
Advanced modalities (paired CBCT + IOS, e.g., PerioAI)	Non-invasive, sub-millimetre 3D digital probing that may complement, and with further validation potentially reduce reliance on, manual probing where equipment exists.	Equipment cost, radiation dose, and concentration in urban centres limit equitable rollout; requires local validation.	[15,16]
Data sovereignty and security	Sovereign data substrate and local hosting support patient data control and the Concept’s sovereignty goals.	Rising cyberattack volume and breach history raise confidentiality and integrity risks for health data.	[25,36,48]

Reference numbers in the Refs. column correspond to the numbered reference list of the main manuscript. Abbreviations: AI, artificial intelligence; CBCT, cone beam computed tomography; EHR, electronic health record; EU, European Union; FDA, U.S. Food and Drug Administration; GPU, graphics processing unit; IoT, Internet of Things; IOS, intraoral scanning.

## Data Availability

No new data were created or analyzed in this study. Data sharing is not applicable to this article.

## Supplementary Materials

The following supporting information can be downloaded at: https://www.mdpi.com/article/10.3390/dj14090615/s1. Supplementary Material S1: Completed PRISMA 2020 27-item checklist [30] with the location of each reporting item in the manuscript; Supplementary Material S2: Completed PRISMA-ScR 20-item checklist with the location of each reporting item in the manuscript; Supplementary Material S3: Full database-specific search strategies (PubMed/MEDLINE, Embase, Scopus, Web of Science, and Cochrane), including verbatim search strings, applied filters, and the number of records retrieved on the date of the last search (30 April 2025); Supplementary Table S4: Complete structured charting of the 29 peer-reviewed empirical and review studies included in the qualitative synthesis (author/year, country, data type, AI method, sample size, validation type, and key reported performance); Supplementary Material S5: Full list of the 40 included information sources, grouped into peer-reviewed empirical and review studies (*n* = 29), regulatory, grey literature, and patent documents (*n* = 11), and background and methodological works cited but not counted among the included sources (*n* = 10). Supplementary Table S6: Mapping of the QUADAS-2, APPRAISE-AI, MI-CLAIM, CONSORT-AI, and SPIRIT-AI critical appraisal tools to each of the 29 included peer-reviewed empirical and review studies, with the rationale where a given tool was not applicable and the key reported finding of each study.
