# Supplementary material for: Bridging the Implementation Gap: Artificial Intelligence in Periodontology from Proof-of-Concept to Clinical Governance—A Systematic Scoping Review with Evidence from Kazakhstan
Source: Dent J (Basel). 2026 Sep 21;14(9):615. doi: 10.3390/dj14090615 (PMC13604993; doi:10.3390/dj14090615)
Supplement: Supplementary file 1 [file dentistry-14-00615-s001.zip › dentistry-4361478-supplementary.pdf]

Supplementary Material S1. PRISMA 2020 Checklist

Section / Topic	Item #	Checklist item	Location in manuscript / comment
**TITLE**	1	Title — Identify the report as a systematic review.	Title: "Bridging the Implementation Gap … A Systematic Scoping Review with Regulatory and Contextual Evidence from Kazakhstan" (title corrected to match the current main-manuscript title; the previous shorter title cited here was a residual cross-file inconsistency from an earlier revision.)
**ABSTRACT**	2	Abstract — See the PRISMA 2020 for Abstracts checklist.	Abstract (Background/Objectives, Methods, Results, Conclusions); structured following PRISMA-A.
**INTRODUCTION**	3	Rationale — Describe the rationale for the review in the context of existing knowledge.	Section 1.1–1.3 (Global burden; State of the art; Research gap).
**INTRODUCTION**	4	Objectives — State the objective(s) the review addresses.	Section 1.5 Research Objectives (three interconnected objectives).
**METHODS**	5	Eligibility criteria — Specify the inclusion and exclusion criteria for the review and how studies were grouped for the syntheses.	Section 2.3 Eligibility Criteria, structured by Population–Concept–Context (PCC) framework.
**METHODS**	6	Information sources — Specify all databases, registers, websites, organisations, reference lists and other sources searched.	Section 2.4 Information Sources (5 databases + WIPO PatentScope + grey-literature sources + citation searching).
**METHODS**	7	Search strategy — Present the full search strategies, including any filters and limits used.	Section 2.5 Search Strategy (Boolean strings, filters); full database-specific search strings in Supplementary Material S3.
**METHODS**	8	Selection process — Specify the methods used to decide whether a study met the inclusion criteria.	Section 2.6 Selection Process (two reviewers, duplicate, Cohen's κ = 0.81; third-reviewer adjudication).
**METHODS**	9	Data collection process — Specify the methods used to collect data from reports.	Section 2.7 Data-Charting (Extraction) Process (a-priori form, duplicate extraction, calibrated on 5 studies).
**METHODS**	10a	Data items — List and define all outcomes for which data were sought.	Section 2.8 Data Items — empirical outcomes: accuracy, sensitivity, specificity, AUC, F1, correlation.
**METHODS**	10b	Data items — List and define all other variables for which data were sought.	Section 2.8 Data Items — bibliographic data, design, dataset, AI architecture, modality, application domain, validation, limitations, ethical/regulatory considerations.
**METHODS**	11	Study risk-of-bias assessment — Specify the methods used to assess risk of bias.	Section 2.9 Critical Appraisal — QUADAS-2 for diagnostic-accuracy studies; APPRAISE-AI, MI-CLAIM, CONSORT-AI,

Section / Topic	Item #	Checklist item	Location in manuscript / comment
			SPIRIT-AI for reporting completeness.
**METHODS**	12	Effect measures — Specify the effect measure(s) used in the synthesis.	Not applicable — descriptive scoping synthesis without statistical pooling (Section 2.10).
**METHODS**	13a	Synthesis methods — Describe the processes used to decide which studies were eligible for each synthesis.	Section 2.10 Synthesis of Results — framework-based thematic synthesis with seven clusters.
**METHODS**	13b	Synthesis methods — Describe any methods required to prepare the data for presentation.	Section 2.10 — performance metrics presented as ranges (min–max); narrative tabulation by theme.
**METHODS**	13c	Synthesis methods — Describe any methods used to tabulate or visually display results.	Section 2.10; Table 1 (empirical studies), Table 2 (regulatory frameworks), and Table 3 (Kazakhstan resources and risks); Figures 1–2.
**METHODS**	13d	Synthesis methods — Describe any methods used to synthesise results and provide a rationale for the choice(s).	Section 2.10 — thematic synthesis chosen given the methodological, clinical and statistical heterogeneity precluding meta-analysis; rationale in Section 2.1.
**METHODS**	13e	Synthesis methods — Describe any methods used to explore possible causes of heterogeneity.	Heterogeneity sources discussed narratively (data scale, external-validation absence, labelling inconsistency, LMIC under-representation) — Section 3.5.
**METHODS**	13f	Synthesis methods — Describe any sensitivity analyses conducted to assess robustness.	Not applicable — descriptive synthesis without quantitative effect estimates.
**METHODS**	14	Reporting bias assessment — Describe any methods used to assess risk of bias due to missing results.	Not applicable to a scoping review of heterogeneous evidence (Section 2.11); no funnel-plot analysis.
**METHODS**	15	Certainty assessment — Describe any methods used to assess certainty in the body of evidence.	Section 2.11 — formal GRADE not applied (PRISMA-ScR-consistent); certainty conveyed narratively with reference to design, dataset size, external validation.
**RESULTS**	16a	Study selection — Describe results of the search and selection process.	Section 3.1 Study Selection — full numbers; Figure 1 PRISMA 2020 flow diagram.
**RESULTS**	16b	Study selection — Cite studies that might appear to meet the inclusion criteria but which were excluded, with reasons.	Figure 1 (numbers and reasons for full-text exclusions); detailed list in Supplementary Material S3.
**RESULTS**	17	Study characteristics — Cite each included study and present its characteristics.	Sections 3.2–3.8 (themes); Tables 1–3; individual studies cited and described.
**RESULTS**	18	Risk of bias in studies — Present assessments of risk of bias for each included study.	Section 3.5 (Data Quality and External Validation) — narrative appraisal summary.

Section / Topic	Item #	Checklist item	Location in manuscript / comment
**RESULTS**	19	Results of individual studies — Present, for all outcomes, for each study, summary statistics.	Sections 3.2–3.4 — quantitative metrics (AUC, accuracy, sensitivity) reported per study.
**RESULTS**	20a	Results of syntheses — For each synthesis, briefly summarise the characteristics and risk of bias of contributing studies.	Each Theme section (3.2–3.8) opens with synthesis narrative.
**RESULTS**	20b	Results of syntheses — Present results of all statistical syntheses.	Not applicable — no statistical synthesis; performance ranges reported.
**RESULTS**	20c	Results of syntheses — Present results of all investigations of possible causes of heterogeneity.	Section 3.5 — heterogeneity sources discussed narratively.
**RESULTS**	20d	Results of syntheses — Present results of all sensitivity analyses.	Not applicable — descriptive synthesis.
**RESULTS**	21	Reporting biases — Present assessments of risk of bias due to missing results.	Not applicable; limitation acknowledged in Section 4.4.
**RESULTS**	22	Certainty of evidence — Present assessments of certainty in the body of evidence.	Section 4.1 and Section 4.4 — certainty conveyed narratively with reference to external-validation limitations.
**DISCUSSION**	23a	Discussion — Provide a general interpretation of the results in the context of other evidence.	Section 4.1 The Dual Reality.
**DISCUSSION**	23b	Discussion — Discuss any limitations of the evidence included in the review.	Section 3.5 (evidence-base limitations) and Section 4.1.
**DISCUSSION**	23c	Discussion — Discuss any limitations of the review processes used.	Section 4.4 Limitations (language, no published Kazakhstan-specific clinical AI studies, evolving regulation).
**DISCUSSION**	23d	Discussion — Discuss implications of the results for practice, policy and future research.	Section 4.2 (six-stage implementation framework) and Section 5 (Conclusions).
**OTHER**	24a	Registration and protocol — Provide registration information for the review.	Section 2.2 Protocol and Registration — protocol developed a priori; PROSPERO does not accept scoping reviews; documented internally and available from corresponding author.
**OTHER**	24b	Registration and protocol — Indicate where the review protocol can be accessed.	Section 2.2 — available from the corresponding author on reasonable request.
**OTHER**	24c	Registration and protocol — Describe and explain any amendments to information provided at registration or in the protocol.	Section 2.2 — two amendments are recorded: (i) minor refinements to the search strings after pilot searching; and (ii) incorporation of additional sources identified after the 30 April 2025 search closure through expert peer review and citation checking rather than database searching. Both are documented in Supplementary Material S3.
**OTHER**	25	Support — Describe sources of financial or non-financial support for the review.	Funding statement: "This research received no external funding."

Section / Topic	Item #	Checklist item	Location in manuscript / comment
**OTHER**	26	Competing interests — Declare any competing interests of review authors.	Conflict of Interest statement: "The authors declare no competing financial or non-financial interests."
**OTHER**	27	Availability of data, code and other materials — Report which of the following are publicly available.	Data Availability Statement; PRISMA flow diagram (Figure 1); checklists in S1, S2; search strings in S3; charted dataset available from corresponding author.

Supplementary Material S2. PRISMA-ScR Checklist (Scoping Reviews)

Section	Item #	Item	Description	Location in manuscript
**TITLE**	1	**Title**	Identify the report as a scoping review.	Title contains "Systematic Scoping Review".
**ABSTRACT**	2	**Structured summary**	Provide a structured summary that includes (as applicable): background, objectives, eligibility criteria, sources of evidence, charting methods, results, and conclusions that relate to the review questions and objectives.	Abstract is structured (Background/Objectives, Methods, Results, Conclusions).
**INTRODUCTION**	3	**Rationale**	Describe the rationale for the review in the context of what is already known. Explain why the review questions / objectives lend themselves to a scoping review approach.	Sections 1.1–1.3; rationale for scoping design specifically in Section 2.1.
**INTRODUCTION**	4	**Objectives**	Provide an explicit statement of the questions and objectives being addressed with reference to their key elements (e.g. population or participants, concepts, and context) or other relevant key elements used to conceptualise the review questions and/or objectives.	Section 1.5 (three objectives); PCC framework in Section 2.3.
**METHODS**	5	**Protocol and registration**	Indicate whether a review protocol exists; state if and where it can be accessed (e.g., a web address); and if available, provide registration information including the registration number.	Section 2.2 — protocol developed a priori; available from corresponding author; PROSPERO does not register scoping reviews.
**METHODS**	6	**Eligibility criteria**	Specify characteristics of the sources of evidence used as eligibility criteria (e.g. years considered, language, publication status), and provide a rationale.	Section 2.3 — inclusion/exclusion with PCC framework; years 2015–2025; English & Russian.
**METHODS**	7	**Information sources**	Describe all information sources in the search (e.g., databases with dates of coverage and contact with authors to identify additional sources), as well as the date the most recent search was executed.	Section 2.4 — 5 databases + WIPO + grey-literature websites + backward citation searching; last search 30 April 2025.
**METHODS**	8	**Search**	Present the full electronic search strategy for at least 1 database, including any limits used, such that it could be repeated.	Section 2.5 — Boolean strings and filters; full per-database strings in Supplementary Material S3.
**METHODS**	9	**Selection of sources of evidence**	State the process for selecting sources of evidence (i.e., screening and eligibility) included in the scoping review.	Section 2.6 — duplicate, independent two-stage screening with consensus and third-reviewer adjudication.
**METHODS**	10	**Data charting process**	Describe the methods of charting data from the included sources of evidence (e.g., calibrated forms or forms that have been tested by the team before their use, and whether data charting was done independently or in duplicate) and any	Section 2.7 — calibrated form piloted on 5 studies; duplicate independent extraction; consensus resolution.

Section	Item #	Item	Description	Location in manuscript
			processes for obtaining and confirming data from investigators.	
**METHODS**	11	**Data items**	List and define all variables for which data were sought and any assumptions and simplifications made.	Section 2.8 Data Items.
**METHODS**	12	**Critical appraisal of individual sources of evidence**	If done, provide a rationale for conducting a critical appraisal of included sources of evidence; describe the methods used and how this information was used in any data synthesis (if appropriate).	Section 2.9 — QUADAS-2 plus AI-specific reporting checklists; findings informed but did not exclude sources.
**METHODS**	13	**Synthesis of results**	Describe the methods of handling and summarising the data that were charted.	Section 2.10 — framework-based thematic synthesis; metrics as ranges; seven clusters.
**RESULTS**	14	**Selection of sources of evidence**	Give numbers of sources of evidence screened, assessed for eligibility, and included in the review, with reasons for exclusions at each stage, ideally using a flow diagram.	Section 3.1 + Figure 1 PRISMA 2020 flow diagram (numbers consistent with text).
**RESULTS**	15	**Characteristics of sources of evidence**	For each source of evidence, present characteristics for which data were charted and provide the citations.	Sections 3.2–3.8; Table 1 (empirical studies), Table 2 (regulatory frameworks), and Table 3 (Kazakhstan resources and risks).
**RESULTS**	16	**Critical appraisal within sources of evidence**	If done, present data on critical appraisal of included sources of evidence (see item 12).	Section 3.5 (Data Quality and External Validation Barriers).
**RESULTS**	17	**Results of individual sources of evidence**	For each included source of evidence, present the relevant data that were charted that relate to the review questions and objectives.	Sections 3.2–3.8 — metric ranges, methodologies, domains.
**RESULTS**	18	**Synthesis of results**	Summarise and / or present the charting results as they relate to the review questions and objectives.	Sections 3.2–3.8 — seven themes; Figure 2 implementation framework.
**DISCUSSION**	19	**Summary of evidence**	Summarise the main results (including an overview of concepts, themes, and types of evidence available), link to the review questions and objectives, and consider the relevance to key groups.	Section 4.1 Dual Reality; Section 4.3 Unique Contribution.
**DISCUSSION**	20	**Limitations**	Discuss the limitations of the scoping review process.	Section 4.4 Limitations.
**DISCUSSION**	21	**Conclusions**	Provide a general interpretation of the results with respect to the review questions and objectives, as well as potential implications and / or next steps.	Section 5 Conclusions; Section 4.2 six-stage implementation framework.
**FUNDING**	22	**Funding**	Describe sources of funding for the included sources of evidence, as well as sources of funding for the scoping review. Describe the role of the funders of the scoping review.	Funding statement: self-funded; no role of external sponsors.

Supplementary Material S3. Full Database-Specific Search Strategies

PubMed/MEDLINE (via NCBI Entrez)

Date searched: 30 April 2025

Filters applied: Publication date 2015/01/01 – 2025/04/30; Languages: English, Russian.

Records retrieved: 10,189

Search strategy:

#1 "artificial intelligence"[MeSH] OR "artificial intelligence"[tiab] OR "machine learning"[MeSH] OR "machine learning"[tiab] OR "deep learning"[tiab] OR "neural network*"[tiab] OR "convolutional neural network*"[tiab] OR "natural language processing"[tiab] OR "vision transformer*"[tiab]

#2 "periodontitis"[MeSH] OR "periodontitis"[tiab] OR "periodontal disease*"[tiab] OR "gingivitis"[MeSH] OR "gingivitis"[tiab] OR "periodontal bone loss"[tiab] OR "alveolar bone loss"[tiab] OR "gingival inflammation"[tiab]

#3 "diagnosis"[tiab] OR "screening"[tiab] OR "prediction"[tiab] OR "monitoring"[tiab] OR "classification"[tiab] OR "governance"[tiab] OR "regulation"[tiab]

#4 #1 AND #2

#5 #4 AND #3

Final #4 with filters: ("2015/01/01"[PDAT] : "2025/04/30"[PDAT]) AND (English[lang] OR Russian[lang])

Embase (via embase.com)

Date searched: 30 April 2025

Filters applied: Date 2015-2025; Languages: English, Russian.

Records retrieved: 6,694

Search strategy:

#1 'artificial intelligence'/exp OR 'machine learning'/exp OR 'deep learning'/exp OR 'neural network'/exp OR 'convolutional neural network':ti,ab,kw OR 'natural language processing':ti,ab,kw OR 'vision transformer':ti,ab,kw

#2 'periodontitis'/exp OR 'periodontal disease'/exp OR 'gingivitis'/exp OR 'alveolar bone loss':ti,ab,kw OR 'periodontal bone loss':ti,ab,kw

#3 'diagnosis':ti,ab,kw OR 'screening':ti,ab,kw OR 'prediction':ti,ab,kw OR 'monitoring':ti,ab,kw OR 'classification':ti,ab,kw OR 'governance':ti,ab,kw OR 'regulation':ti,ab,kw

#4 #1 AND #2

#5 #4 AND #3 AND [2015-2025]/py AND ([english]/lim OR [russian]/lim)

Web of Science Core Collection

Date searched: 30 April 2025

Filters applied: Timespan 2015-2025; Document types: Article, Review; Languages: English, Russian.

Records retrieved: 8,412

Search strategy:

#1 TS=("artificial intelligence" OR "machine learning" OR "deep learning" OR "neural network*" OR "convolutional neural network*" OR "natural language processing" OR "vision transformer*")

#2 TS=("periodontitis" OR "periodontal disease*" OR "gingivitis" OR "periodontal bone loss" OR "alveolar bone loss" OR "gingival inflammation")

#3 TS=("diagnosis" OR "screening" OR "prediction" OR "monitoring" OR "classification" OR "governance" OR "regulation")

#4 #1 AND #2

#5 #4 AND #3 — refined by 2015–2025; LANGUAGES (ENGLISH OR RUSSIAN); DOCUMENT TYPES (ARTICLE OR REVIEW).

Cochrane Library (CENTRAL + CDSR)

Date searched: 30 April 2025

Filters applied: Date 2015–2025.

Records retrieved: 5,457

Search strategy:

#1 ("artificial intelligence" OR "machine learning" OR "deep learning" OR "neural network*" OR "convolutional neural network*"):ti,ab,kw

#2 ("periodontitis" OR "periodontal disease*" OR "gingivitis" OR "periodontal bone loss" OR "alveolar bone loss"):ti,ab,kw

#3 #1 AND #2 in Trials and Cochrane Reviews; Publication Year from 2015 to 2025.

Scopus (via Elsevier)

Date searched: 30 April 2025

Filters applied: PUBYEAR > 2014 AND PUBYEAR < 2026; LIMIT-TO (LANGUAGE, "English" OR "Russian").

Records retrieved: 23,382

Search strategy:

TITLE-ABS-KEY (("artificial intelligence" OR "machine learning" OR "deep learning" OR "neural network*" OR "convolutional neural network*" OR "natural language processing" OR "vision transformer*") AND ("periodontitis" OR "periodontal disease*" OR "gingivitis" OR "periodontal bone loss" OR "alveolar bone loss" OR "gingival inflammation")) AND PUBYEAR > 2014 AND PUBYEAR < 2026 AND (LIMIT-TO (LANGUAGE,"English") OR LIMIT-TO (LANGUAGE,"Russian"))

WIPO PatentScope

Date searched: 30 April 2025

Filters applied: Filing/publication year 2015–2025.

Records retrieved: Variable (top 10 retrieved for screening). Two pending applications later granted as RU2841194 and RU2850170 were provisionally located at this stage, but neither had been granted/publicly citable as a granted patent document by 30 April 2025 (verified grant/publication dates: 3 June 2025 and 5 November 2025, respectively); both are therefore reported as revision additions rather than original-search inclusions (see Supplementary Material S5).

Search strategy:

FP:("artificial intelligence" OR "machine learning" OR "neural network") AND FP:("periodont*" OR "gingiv*") AND DP:[2015 TO 2025]

Grey-literature and regulatory sources (hand-searched)

Date searched: 30 April 2025

Filters applied: Latest official version retrievable.

Records retrieved: 8 documents screened (7 regulatory/policy documents counted among the included grey/regulatory sources, plus the WHO Global Oral Health Status Report cited as background), giving 7 regulatory sources identified by the original search (no patents were yet grantable as of 30 April 2025; see WIPO PatentScope note above). Four further regulatory/grey-literature/patent sources—Kazakhstan Law No. 230-VIII 'On Artificial Intelligence' (enacted 17 November 2025); the NIT National Supercomputer (Alem.Cloud) infrastructure source (launched July 2025); and two granted Russian patents, RU2841194 (granted 3 June 2025) and RU2850170 (granted 5 November 2025), both provisionally located as pending applications during the original patent search but not yet grantable as of search closure—were added during revision to capture binding legislation, national AI infrastructure, and the two now-granted patents, giving 11 regulatory, grey-literature, and patent documents among the 40 included information sources (7 from the original search + 4 added during revision).

Search strategy:

Searched portals: who.int, fda.gov, eur-lex.europa.eu, unesco.org, imdrf.org, adilet.zan.kz, online.zakon.kz.
Search terms: ("artificial intelligence" OR "machine learning") AND ("medical device" OR "software as a medical device" OR "health" OR "healthcare").

Documents retrieved and screened against eligibility criteria: WHO Global Oral Health Status Report (2022); WHO Ethics and Governance of AI for Health (2021); EU AI Act – Regulation (EU) 2024/1689; FDA SaMD Action Plan (2021); UNESCO Recommendation on the Ethics of AI (2021); Kazakhstan Government Resolution No. 592 of 24 July 2024; Order of the Ministry of Healthcare of the Republic of Kazakhstan No. ҚР ДСМ-39 of 12 May 2021; Order of the Ministry of Digital Development of the Republic of Kazakhstan No. 199/НҚ of 21 June 2023.

Refinements to the search strategy after pilot searching

After pilot searching in PubMed/MEDLINE (1 March 2025), the following terms were added to Block #1 (AI technology) to improve recall: "convolutional neural network*", "natural language processing", and "vision transformer*". One further amendment is recorded: after the search closure date of 30 April 2025, ten additional sources meeting the pre-specified eligibility criteria but published after this date were identified through expert peer review and citation checking, rather than through a repeat database search, and incorporated during revision (six peer-reviewed empirical/review/interpretive studies and four regulatory/grey-literature/patent documents; see Section 2.2 and Section 3.1 of the main manuscript). These ten sources are reported as records identified by other methods in the PRISMA flow diagram (Figure 1) and are itemised with exact publication dates in Supplementary Material S5. This was a targeted, citation-triggered amendment rather than a full re-execution of the search across all five databases and the patent registry to a single new closure date; this scope limitation is stated explicitly in Section 4.4 of the main manuscript. No other amendments were made to the protocol.

Supplementary Table S4. Complete structured charting of the 29 peer-reviewed empirical and review studies included in the qualitative synthesis (author/year, country, data type, AI method, sample size, validation type, and key reported performance)

Complete structured charting of all 29 peer-reviewed empirical, review, and companion interpretive sources included in the qualitative synthesis (author/year, country, input data type, AI method/architecture, sample size, validation type, and key reported performance). All 29 peer-reviewed empirical and review sources informing the synthesis are charted here (the 29 rows of this table correspond to references [6–22], [30–34], [37–39], and [45–46,48–49] of the main manuscript); review-type and protocol sources are indicated as evidence-synthesis entries for which primary diagnostic validation is not applicable. A shorter representative subset spanning the main application domains is reproduced in Table 1 of the main manuscript. Together with the 11 regulatory, grey-literature, and patent documents (listed and characterised in Table 2 of the main manuscript and in Supplementary Material S3), these 29 studies constitute the 40 information sources included in the review. The regulatory, grey-literature, and patent documents are not charted in this table.

Author (Year)	Country	Data type	AI method / architecture	Sample size	Validation type	Key reported performance
Krois et al. (2019) [11]	Germany	Panoramic radiograph segments	Deep feed-forward CNN (7-layer)	2,001 image segments	Internal (cross-validation)	Accuracy 0.81 for PBL; matched mean of 6 dentists (0.76)
Dujic et al. (2023) [12]	Germany	Periapical radiographs	Vision Transformer (ViT)	Periapical radiograph dataset	Internal	Accuracy 83.4–85.2%; AUC 0.899–0.918 (lower anterior AUC up to 0.970)
Kim et al. (2022) [13]	South Korea	Panoramic radiographs	Faster R-CNN + Inception + ResNet	Hospital panoramic dataset	Internal (clinical workflow)	Real-time multi-disease detection (5 oral pathologies); separate model per disease
Li et al. (2025) [14]	China	Intraoral scans (IOS)	GC-U-Net (global-context attention)	IOS gingival image dataset	Internal	r = 0.836 (Sulcus Bleeding Index); r = 0.618 (Bleeding Index)
Tan et al. (2025) [15]	China (multicentre)	Intraoral scans (IOS) + CBCT	Full-stack DL (IOS segmentation + CBCT segmentation + multimodal fusion + digital probing)	2,507 patients (multicentre cohorts)	Internal and external	Automated 3D gingiva–bone distance; digital probing error 0.040 mm; tooth-level volumetric diagnosis
Wu & Tsoi (2025) [16]	China (Hong Kong) (commentary)	IOS + CBCT multimodal (commentary)	Commentary / perspective	N/A (commentary)	N/A (commentary)	IOS–CBCT multimodal pipelines can replace manual probing and advance non-invasive diagnosis
Alalharith et al. (2020) [17]	Saudi Arabia	Intraoral photographs (orthodontic)	Faster R-CNN + ResNet-50	Orthodontic patient images	Internal	Accuracy 77.12% for early gingivitis detection
Kurt-Bayrakdar et al. (2024) [42]	Turkey	Panoramic radiographs	Two-stage object detection (DL)	Retrospective panoramic dataset	Internal	High sensitivity for horizontal/vertical bone loss patterns and furcation; stage III/IV
Xue et al. (2024) [43]	China	Panoramic radiographs	DL pipeline (segmentation + bone-level quantification)	Panoramic dataset	Internal	High concordance with specialist staging
Bashir et al. (2022) [18]	USA / Taiwan	NHANES + Taiwanese national data	10 ML algorithms (comparative)	Nationally representative datasets	External (cross-national)	High internal accuracy; significant external-validation performance drop
Deng et al. (2024) [19]	China (Shanghai / Hong Kong)	Patient-reported + salivary MMP-8/Hb	Random forest, gradient boosting (multiclass)	Community screening cohort	Internal	Discriminated health/gingivitis/Stage I–II/Stage III–IV
Deng et al. (2022) [37]	China	Saliva / oral-rinse biomarkers	Point-of-care biomarker (aMMP-8) analysis	Diagnostic-accuracy cohort	Internal	Reflected current disease activity vs historical destruction
Tonetti et al. (2020) [20]	Multinational (IoT network)	IoT toothbrush + patient-reported	IoT observational monitoring; mixed-effects/stepwise regression	Supportive periodontal care population	N/A (non-ML prospective observational study)	Self-reported bleeding on brushing predicted bleeding on probing

Author (Year)	Country	Data type	AI method / architecture	Sample size	Validation type	Key reported performance
Yan et al. (2025) [30]	UK	Periodontal + systemic biomarkers	ML (association modelling)	Clinical/biomarker dataset	Internal	Quantified PISA–CRP and systemic-marker associations
Khubrani et al. (2025) [21]	Multinational (review)	2D dental radiographs (review)	Systematic review/meta-analysis (APPRAISE-AI)	Studies 1990–2024	N/A (evidence synthesis)	Pooled high sensitivity; marked heterogeneity; few external validations
Ferrara et al. (2025) [7]	Italy (review)	Radiographic DL (review)	Systematic review (QUADAS-2)	13 studies appraised	N/A (evidence synthesis)	Accuracy range 73.0–98.6%; most lacking external validation
Scott et al. (2023) [6]	UK (review)	Mixed AI in periodontology (review)	Scoping review	50 included studies	N/A (evidence synthesis)	Heterogeneous reporting outcomes across studies
Umer et al. (2024) [22]	Pakistan (LMIC review)	AI in dentistry, LMICs (review)	Scoping review	25 of 1,587 records (LMIC)	N/A (evidence synthesis)	Severe underrepresentation of LMIC/transitional economies
Shuto et al. (2025) [44]	Japan (review)	Facial scans / digital records (review)	Scoping review	Facial-scan literature	N/A (evidence synthesis)	Absence of standardised multimodal integration protocols
Jundaeng et al. (2024) [8]	Thailand (review)	AI in periodontal diagnosis (review)	Narrative/scoping review	AI-diagnosis literature	N/A (evidence synthesis)	AI-powered innovations span the full periodontal diagnostic pathway
Jafer et al. (2025) [9]	Saudi Arabia (review)	AI models for periodontal diagnosis (review)	Systematic review	Included AI-model studies	N/A (evidence synthesis)	Appraised AI performance for diagnosis, classification, and prediction of periodontal diseases
Revilla-Léon et al. (2023) [10]	USA / Spain (review)	AI for gingivitis/periodontal diagnosis (review)	Systematic review	Included diagnostic-AI studies	N/A (evidence synthesis)	AI models for diagnosing gingivitis and periodontal disease; heterogeneous performance
Kengne Talla et al. (2024) [40]	Canada (review protocol)	Teledentistry access/quality (review protocol)	Overview of systematic reviews / meta-analyses (protocol)	Protocol (no pooled n)	N/A (protocol)	Evidence-supported potential of teledentistry to improve access and quality of oral care
Schwendicke et al. (2021) [49]	Germany (review)	Cephalometric radiographs (review)	DL landmark detection; systematic review/meta-analysis	Included DL studies	N/A (evidence synthesis)	Pooled DL accuracy for cephalometric landmark detection; methodological heterogeneity
Gusev et al. (2022) [38]	Russian Federation (methodology)	ML model development/validation reporting	Methodological framework (MI-CLAIM-aligned)	Methodological guidance	N/A (methodology)	Standardised reporting requirements for ML model creation and validation
Sagatov et al. (2022) [41]	Kazakhstan	Population oral-health survey	Cross-sectional epidemiological survey	National survey sample	Observational (survey)	Documented unmet oral-health care needs in Kazakhstan
Issilbayeva et al. (2025) [31]	Kazakhstan	Oral-microbiome / clinical data	Microbiome association analysis	Adolescent cohort	Observational	Oral-microbiome patterns of dental caries in Kazakhstani adolescents
Sarakbi et al. (2025) [32]	UAE (review)	AI in periodontal maintenance (review)	Scoping review	Maintenance-AI literature	N/A (evidence synthesis)	AI supports precision monitoring and personalised supportive periodontal care
Kengne Talla et al. (2023) [39]	Canada (review protocol)	Teledentistry implementation barriers (review protocol)	Mixed-studies review (protocol)	Protocol (no pooled n)	N/A (protocol)	Framework of professional, organisational, and infrastructure barriers to teledentistry

Abbreviations: AUC, area under the ROC curve; CBCT, cone-beam computed tomography; CNN, convolutional neural network; CRP, C-reactive protein; DL, deep learning; Hb, haemoglobin; IOS, intraoral scanning; IoT, Internet of Things; LMIC, low- and middle-income country; ML, machine learning; MMP-

8, matrix metalloproteinase-8; NHANES, National Health and Nutrition Examination Survey; PBL, periodontal bone loss; PISA, periodontal inflamed surface area; ViT, Vision Transformer. N/A indicates an evidence-synthesis (review) source for which primary validation is not applicable.

Supplementary Material S5. Full list of the 40 included information sources

The 40 information sources meeting the inclusion criteria comprise 29 peer-reviewed empirical and review studies (Group A, charted in full in Supplementary Table S4) and 11 regulatory, grey-literature, and patent documents (Group B, characterised in Table 2 of the main manuscript). Of these 40, 30 (23 in Group A + 7 in Group B) were identified by the original search, which closed 30 April 2025, and 10 (6 in Group A + 4 in Group B) were identified after that date through expert peer review, citation checking, and reviewer requests during revision (Section 2.2 and Section 3.1 of the main manuscript); the origin and, for the revision-added sources, the verified publication/grant date of every item are given below. For transparency, the nine additional background and methodological works that are cited in the manuscript but were not treated as included information sources are listed separately (Group C); two of these nine (refs. 26 and 27) are also noted below as having been published after 30 April 2025, even though they are not counted among the 40 included sources. The included sources (Groups A and B, 40 items) plus the nine Group C background works account for the 49 entries in the numbered reference list. Reference numbers refer to the main-manuscript reference list.

Group A. Peer-reviewed sources satisfying inclusion criteria (n = 29; charted in full in Supplementary Table S4). This group is heterogeneous by design (Section 2.3): it comprises primary AI/ML empirical studies, evidence-synthesis (systematic/scoping) reviews, one companion commentary, one non-AI biomarker-validation study, one methodological/reporting-standards source, two non-AI teledentistry review protocols, and two Kazakhstan-specific population-context studies. It should not be read as 29 primary AI/clinical-performance studies; the formally appraisable subset comprises the 12 studies identified in Supplementary Table S6; Tonetti et al. (2020) [20] is retained separately as contextual, non-ML IoT evidence.

[6] Scott et al. (2023) — original search. [7] Ferrara et al. (2025) — original search. [8] Jundaeng et al. (2024) — original search. [9] Jafer et al. (2025) — ADDED DURING REVISION (published 18 December 2025; identified by citation checking, not by the original search). [10] Revilla-León et al. (2023) — original search. [11] Krois et al. (2019) — original search. [12] Dujic et al. (2023) — original search. [13] Kim et al. (2022) — original search. [14] Li et al. (2025) — original search (epub 27 January 2025, before search closure). [15] Tan et al. (2025) — ADDED DURING REVISION (published June 2025; PerioAI primary study). [16] Wu & Tsoi (2025) — ADDED DURING REVISION (published June 2025; companion commentary to [15], single pre-specified exception per Section 2.3). [17] Alalharith et al. (2020) — original search. [18] Bashir et al. (2022) — original search. [19] Deng et al. (2024) — original search. [20] Tonetti et al. (2020) — original search. [21] Khubrani et al. (2025) — original search (epub 5 December 2024, print February 2025, before search closure). [22] Umer et al. (2024) — original search. [42] Kurt-Bayrakdar et al. (2024) — original search. [43] Xue et al. (2024) — original search. [37] Deng et al. (2022) — original search. [30] Yan et al. (2025) — ADDED DURING REVISION (epub 23 July 2025; identified by citation checking, not by the original search). [44] Shuto et al. (2025) — original search (published 3 April 2025, before search closure). [40] Kengne Talla et al. (2024) — original search. [49] Schwendicke et al. (2021) — original search. [38] Gusev et al. (2022) — original search. [41] Sagatov et al. (2022) — original search. [31] Issilbayeva et al. (2025) — ADDED DURING REVISION (published 9 December 2025; identified by expert peer review, not by the original search). [32] Sarakbi et al. (2025) — ADDED DURING REVISION (published 14 May 2025; identified by citation checking, not by the original search). [39] Kengne Talla et al. (2023) — original search. Summary: 23 of the 29 Group A sources were identified by the original search; 6 ([9],[15],[16],[30],[31],[32]) were added during revision, all published after 30 April 2025.

Group B. Regulatory, grey-literature, and patent documents (n = 11; characterised in Table 2). 7 of these 11 were identified by the original search; 4 were added during revision (all published after 30 April 2025).

Regulatory and policy documents (n = 9): [23] EU AI Act, Regulation (EU) 2024/1689 — original search. [24] U.S. FDA SaMD Action Plan — original search. [25] Kazakhstan Government Resolution No. 592 (2024–2029 AI Development Concept) — original search. [45] WHO Ethics and Governance of AI for Health (2021) — original search. [46] UNESCO Recommendation on the Ethics of AI (2021) — original search. [35] Kazakhstan Law No. 230-VIII 'On Artificial Intelligence' (2025) — ADDED DURING REVISION (enacted 17 November 2025). [36] NIT National Supercomputer (Alem.Cloud) infrastructure source (2025) — ADDED DURING REVISION (launched July 2025). [47] Order No. 199/НҚ of the Ministry of Digital Development (2023) — original search. [48] Order No. ҚР ДСМ-39 of the Ministry of Healthcare (2021) — original search. Granted patents (n = 2): [33] Patent RU2841194 — ADDED DURING REVISION (granted 3 June 2025; provisionally located as a pending application during the original patent search but not grantable/publicly citable as a granted patent until after search closure). [34] Patent RU2850170 — ADDED DURING REVISION (granted 5 November 2025; provisionally located as a pending application during the original patent search but not grantable/publicly citable as a granted patent until after search closure; no publication date for this patent was previously stated in the manuscript — it has now been verified and is documented here). Summary: 7 of the 9 regulatory/policy documents were identified by the original search ([35] and [36] were added during revision); both patents were added during revision.

Group C. Background and methodological works cited but not counted among the included sources (n = 10). These are cited for context or state-of-the-art positioning and are not part of the 40 included information sources; two of them (refs. 26 and 27) were nonetheless published after the 30 April 2025 search closure, which is noted here for full transparency even though it does not affect the n = 40 PRISMA count.

[1] Tonetti et al. (2017), global burden of periodontal diseases; [2] WHO Global Oral Health Status Report (2022); [3] Sanz et al. (2020), periodontitis and cardiovascular disease consensus; [4] Papapanou et al. (2018), 2017 World Workshop classification; [5] Schwendicke et al. (2020), AI in dentistry — chances and challenges; [26] Chatzopoulos et al. (2025), clinical applications of AI in periodontology (scoping review, cited for state-of-the-art positioning in Section 1.4 and not charted as an included source; published online 10 June 2025 — after the 30 April 2025 search closure, though this does not affect the n = 40 count since it is not an included source); [27] Roy et al. (2025), AI for diagnosis of periodontal and peri-implant diseases (narrative review, cited for state-of-the-art positioning in Section 1.4 and not charted as an included source; published online 4 June 2025 — after the 30 April 2025 search closure, though this does not affect the n = 40 count since it is not an included source); [28] Page et al. (2021), PRISMA 2020 statement; [29] Tricco et al. (2018), PRISMA-ScR extension; [50] PRISMA 2020 Checklist (2020), the completed reporting checklist accompanying the PRISMA 2020 statement, provided as Supplementary Material S1.

Reconciliation: Group A (29) + Group B (11) = 40 included information sources; 40 + Group C (10) = 50 numbered reference-list entries. Of the 40 included sources, 30 (23 Group A + 7 Group B) were identified by the original search (closed 30 April 2025) and 10 (6 Group A + 4 Group B) were added during revision, all published after that date (Section 2.2 and Section 3.1 of the main manuscript). This 10-source revision update was a targeted, citation- and peer-review-triggered amendment and not a full re-execution of the systematic search across all five databases and the patent registry to a single new closure date; formal search completeness beyond 30 April 2025 is therefore not claimed (Section 4.4 of the main manuscript).

Supplementary Table S6. Mapping of the QUADAS-2, APPRAISE-AI, MI-CLAIM, CONSORT-AI, and SPIRIT-AI critical appraisal tools to each of the 29 included peer-reviewed empirical and review studies, with the rationale where a given tool was not applicable and the key reported finding of each study (Section 2.9). Of the 29 studies, 12 are ones for which a primary-study appraisal tool was judged applicable — 11 primary AI/ML diagnostic-accuracy, predictive, or association-modelling studies plus one non-AI point-of-care biomarker diagnostic-accuracy study assessed under QUADAS-2 on design grounds (Deng et al. 2022 [37]) — and 17 evidence-synthesis, protocol, commentary, methodological, observational IoT, or population-context sources for which these tools do not apply, with the rationale given in each case (12 + 17 = 29). This is not a set of five homogeneous, interchangeable 'critical appraisal tools' applied uniformly; each tool targets a different design and reporting dimension, and the applicable tool(s) vary by study type as shown below. Eleven of the 12 formally appraisable rows below carry an AI-assisted draft appraisal, produced by locating and reading each published study and forming source-grounded QUADAS-2/APPRAISE-AI/MI-CLAIM judgements; this draft is explicitly pending confirmation by reviewers A.C. and A.A. and should not be read as their completed independent dual appraisal until they have reviewed, confirmed, or amended it. One row (Xue et al. 2024 [43]) could not be drafted at all because full-text access was unavailable, and remains an open item pending institutional access.

Consistent with Section 2.9, QUADAS-2 and APPRAISE-AI were applied to primary AI diagnostic-accuracy studies; MI-CLAIM (machine-learning model reporting) was applied to primary ML prediction/classification studies; QUADAS-2 was also judged applicable to Deng et al. (2022) [37], a non-AI point-of-care biomarker diagnostic-accuracy study, on design grounds (index test vs reference standard), even though it is not itself an AI/ML source, and it is accordingly counted among the 12 formally appraisable studies rather than among the 17 N/A sources; and CONSORT-AI (reports of randomised trials of AI interventions) and SPIRIT-AI (protocols for such trials) were considered but were not triggered by any of the 29 included studies, because none was a randomised trial of an AI intervention or an AI-specific trial protocol. Evidence-synthesis sources (systematic/scoping reviews and meta-analyses), non-AI primary studies, a methodological/guidance paper, a companion commentary, and non-AI systematic-review protocols were, by design, not eligible for these primary-study appraisal tools; where a cited source's own review methodology employed one of these tools (e.g., Khubrani et al. 2025 used APPRAISE-AI; Ferrara et al. 2025 used QUADAS-2 within their own reviews), this is noted for transparency but is distinct from the appraisal of that source as an included item in the present review. The 'Domain-level result, main finding, and impact on synthesis' column below currently reports an AI-assisted draft judgement for 11 of the 12 formally appraisable studies, produced by locating and reading each published paper and applying the relevant tool's criteria directly to what that paper reports (with source citations and explicit access-limitation notes in each case); it is not yet the reviewers' own independent judgement. Reviewers A.C. and A.A. are to independently review, confirm, or amend each drafted row — resolving any disagreement between themselves — before this column is treated as final; only Xue et al. (2024) [43] could not be drafted at all, because full-text access was unavailable, and remains marked as not yet appraisable. For all other (N/A) rows, the reason the tool does not apply is stated directly and no appraisal judgement is implied.

Author (Year) [Ref]	Study ID / design	Tool and version (N/A with reason if not applicable)	Basis / rationale	Domain-level result, main finding, and impact on synthesis
Krois et al. (2019) [11]	Primary AI diagnostic-accuracy study (imaging)	QUADAS-2; APPRAISE-AI	Primary DL diagnostic study with reference-standard comparison	AI-assisted draft appraisal (pending confirmation by A.C./A.A.; not their independent judgement). QUADAS-2: high risk of bias for the index test (no independent hold-out set, cross-validation only) and unclear/high for patient selection (single-clinic, single-device, n=85). APPRAISE-AI estimated total ~59/100 (moderate) — an a priori sample-size calculation and candid limitations are genuine strengths, offset by the absence of an independent test set and no code release. Main risk: internal-validation-only design may overstate generalizable accuracy. Impact on synthesis: down-weight relative to externally validated studies. Source: Krois et al., Sci Rep 2019;9:8495 (DOI 10.1038/s41598-019-44839-3, open access).

Author (Year) [Ref]	Study ID / design	Tool and version (N/A with reason if not applicable)	Basis / rationale	Domain-level result, main finding, and impact on synthesis
Dujic et al. (2023) [12]	Primary AI diagnostic-accuracy study (imaging)	QUADAS-2; APPRAISE-AI	Primary DL diagnostic study with reference-standard comparison	AI-assisted draft appraisal (pending confirmation; citation identity should be reconfirmed by authors — a closely related companion paper from the same dataset/group exists, see extraction-sheet notes). QUADAS-2: high risk of bias for patient selection and reference standard (ground truth = dentist visual categorisation of the same radiographs, only fair inter-rater kappa 0.45–0.48, not clinical attachment-loss/probing). APPRAISE-AI estimated total ~47/100 (moderate-low): no external validation, no clinician comparison. Main risk: reference standard substitutes subjective radiographic categorisation for the accepted clinical definition. Impact on synthesis: treat as lower-certainty technical-feasibility evidence, not clinical diagnostic-accuracy evidence. Source: likely Dujic et al., Diagnostics 2023;13(23):3562 (DOI 10.3390/diagnostics13233562).
Kim et al. (2022) [13]	Primary AI diagnostic-accuracy study (imaging, multi-disease)	QUADAS-2; APPRAISE-AI	Primary DL diagnostic study with reference-standard comparison	AI-assisted draft appraisal (pending confirmation). QUADAS-2: high risk of bias for patient selection (case-enriched, non-consecutive sampling across 30–50 clinics) and reference standard (rater qualifications/reliability not reported). APPRAISE-AI estimated total ~40/100 (low-moderate): no external validation, no clinician-comparison arm, no code/data availability. Main risk: unblinded, enrichment-biased sampling with no external validation. Impact on synthesis: weight as low-to-moderate certainty, illustrative-feasibility evidence only. Source: Kim et al., JMIR Med Inform 2022;10(10):e38640 (DOI 10.2196/38640).
Li et al. (2025) [14]	Primary AI diagnostic-accuracy study (imaging)	QUADAS-2; APPRAISE-AI	Primary DL diagnostic study with reference-standard comparison	AI-assisted draft appraisal (pending confirmation). QUADAS-2: high risk of bias for the reference standard in the larger external cohort (n=760; reference derived from the same radiographs being classified — a circularity risk); a second external cohort (n=382) used an independent clinical exam and is low risk. APPRAISE-AI estimated total ~71/100 (moderate-high): strong multicentre external validation and masked specialist comparison (AUROC 94.2% AI vs 85.6% specialists). Main risk: partial reference-standard circularity in the largest external cohort. Impact on synthesis: moderate-to-high-quality evidence overall; down-weight the circular-reference-standard

Author (Year) [Ref]	Study ID / design	Tool and version (N/A with reason if not applicable)	Basis / rationale	Domain-level result, main finding, and impact on synthesis
				comparison specifically. Source: Li et al., npj Digit Med 2025 (DOI 10.1038/s41746-025-02077-0, open access).
Tan et al. (2025) [15]	Primary AI diagnostic-accuracy study (multimodal imaging, internal + external validation)	QUADAS-2; APPRAISE-AI	Primary DL diagnostic study with reference-standard comparison; added during revision (published June 2025)	AI-assisted draft appraisal (pending confirmation). QUADAS-2: high risk of bias for patient selection (retrospective, convenience-pooled, mixed clinical indications); reference-standard/annotator reliability unclear (6 manual annotators, reliability not reported; clinical-probing comparison limited to a 37-patient subset). APPRAISE-AI estimated total ~60/100 (moderate): genuine internal+external validation with stratified robustness analyses is a strength; reproducibility limited (inference code academic-use only). Main risk: non-consecutive, mixed-indication sampling and unreported annotator reliability. Impact on synthesis: moderate-quality evidence, down-weighted for reference-standard/annotation uncertainty. Source: Tan et al., Cell Rep Med 2025 (DOI 10.1016/j.xcrm.2025.102186, open access).
Wu & Tsoi (2025) [16]	Companion commentary (non-primary, interpretive)	N/A – not a primary study; QUADAS-2/APPRAISE-AI/MI-CLAIM/CONSORT-AI/SPIRIT-AI not applicable	Interpretive commentary on an included primary study (Tan et al. 2025 [15]); included solely for interpretive synthesis under the single pre-specified exception (Section 2.3); added during revision (published June 2025)	Not formally appraised. Interpretive commentary, not an empirical study; no independent appraisal target. Cited as evidence-map/contextual information only; not used as clinical-performance evidence (Section 2.10 stratification).
Alalharith et al. (2020) [17]	Primary AI diagnostic-accuracy study (imaging)	QUADAS-2; APPRAISE-AI	Primary DL diagnostic study with reference-standard comparison	AI-assisted draft appraisal (pending confirmation). Note: this study addresses gingivitis (not periodontitis) in orthodontic patients only — a narrower scope than periodontology broadly. QUADAS-2: high risk of bias for patient selection (n=47, single centre) and flow/timing (post-hoc image exclusions without full accounting). APPRAISE-AI estimated total ~36/100 (low-moderate): small single-centre dataset, no external validation, weak recall (41.75%). Main risk: small, non-externally-validated, narrow-scope proof-of-concept. Impact on synthesis: low-to-moderate quality, exploratory evidence only; should not be treated as evidence for periodontitis diagnostic accuracy specifically. Source: Alalharith et al., Int J Environ Res Public Health 2020;17(22):8447 (DOI 10.3390/ijerph17228447, open access).
Kurt-Bayrakdar et al. (2024) [42]	Primary AI diagnostic-accuracy study (imaging)	QUADAS-2; APPRAISE-AI	Primary DL diagnostic study with reference-standard comparison	AI-assisted draft appraisal (pending confirmation). QUADAS-2: high risk of bias and

Author (Year) [Ref]	Study ID / design	Tool and version (N/A with reason if not applicable)	Basis / rationale	Domain-level result, main finding, and impact on synthesis
				applicability concern for patient selection (single-centre/single-scanner, disease-only sample, no healthy/mixed-severity spectrum). APPRAISE-AI estimated total ~48/100 (moderate-low): markedly variable performance across sub-tasks (AUC 0.95 for total bone loss vs near-chance accuracy 0.51 for vertical bone loss), no external test set. Main risk: disease-only sample creates spectrum bias; weak performance on vertical bone-loss/furcation subtasks. Impact on synthesis: low-to-moderate certainty, proof-of-concept evidence only. Source: Kurt-Bayrakdar et al., BMC Oral Health 2024 (DOI 10.1186/s12903-024-03896-5, open access).
Xue et al. (2024) [43]	Primary AI diagnostic-accuracy study (imaging)	QUADAS-2; APPRAISE-AI	Primary DL diagnostic study with reference-standard comparison	NOT YET APPRAISABLE. Full text of this paper could not be retrieved through any accessible route during preparation of this appraisal (publisher paywall; PubMed/Europe PMC/ResearchGate all blocked automated access; no open-access mirror or preprint found). Only bibliographic identity is reasonably established (moderate-high confidence via Crossref: Xue, Chen, Sun, “Deep learning method to automatically diagnose periodontal bone loss and periodontitis stage in dental panoramic radiograph,” J Dent 2024;150:105373, DOI 10.1016/j.jdent.2024.105373), and this has not been cross-checked against this manuscript's own citation. Providing QUADAS-2/APPRAISE-AI ratings without the Methods section would mean inventing rationale, which has not been done. This row requires institutional full-text access (or the authors' own copy of the paper) before it can be appraised at all, and remains the one outstanding blocking item in this table.
Bashir et al. (2022) [18]	Primary AI predictive-modelling study (non-imaging, structured/tabular ML; external cross-national validation)	MI-CLAIM	Primary ML prediction study with external validation; QUADAS-2/APPRAISE-AI not applicable (non-imaging predictive model without an index-test-vs-reference-standard diagnostic design)	AI-assisted draft appraisal (pending confirmation). MI-CLAIM: data-partitioning fully reported and unusually rigorous — genuine cross-country external validation (Taiwan n=3453 / US n=3685), rare in this literature; other sections partially reported at abstract level only (full text blocked by publisher access restrictions). Main finding: external (cross-country) performance “dropped off drastically” relative to internal validation. Impact on synthesis: strong evidence of poor cross-population generalisability of periodontitis ML models; supports a cautious synthesis conclusion about external validity.

Author (Year) [Ref]	Study ID / design	Tool and version (N/A with reason if not applicable)	Basis / rationale	Domain-level result, main finding, and impact on synthesis
				Source: Bashir et al., J Clin Periodontol 2022;49(10):958–969 (DOI 10.1111/jcpe.13692, open access).
Deng et al. (2024) [19]	Primary ML multiclass screening study (patient-reported data + salivary biomarkers)	MI-CLAIM; QUADAS-2 (screening-accuracy elements)	Primary ML screening study combining non-clinical parameters and biomarkers	AI-assisted draft appraisal (pending confirmation). MI-CLAIM: study design and optimisation partially reported (random-forest model, 9 predictors, reference = full-mouth exam per the 2017 classification); data partitioning appears internal-only (external validation explicitly flagged by the authors themselves as not yet done). QUADAS-2 screening-accuracy elements: reference standard low-unclear risk; patient selection/index test/flow-timing unclear (not confirmable from the accessible abstract). Main risk: no confirmed external validation. Impact on synthesis: low-to-moderate confidence primary evidence; the high reported AUC (>0.94) should not be treated as externally validated pending full-text confirmation. Source: Deng et al., J Clin Periodontol 2024;51(12):1547–1560 (DOI 10.1111/jcpe.13856).
Deng et al. (2022) [37]	Non-AI point-of-care biomarker diagnostic-accuracy study	QUADAS-2 (design-appropriate); APPRAISE-AI/MI-CLAIM not applicable (not an AI/ML model)	Non-AI diagnostic-accuracy validation study directly underpinning the biomarker input used by Deng et al. 2024 [19]; included as contextual/methodological evidence per Section 2.3, not as an AI-model source	AI-assisted draft appraisal (pending confirmation). QUADAS-2: reference standard and flow/timing judged low risk (accepted 2017-classification clinical reference; same-visit sequential sampling); patient selection and index-test blinding unclear. Reported accuracy (best-performing sample): sensitivity 80.3%, specificity 67.8%, AUROC 0.740. Main risk: unclear blinding/selection could inflate the reported accuracy figures. Impact on synthesis: as this table's own framework specifies, this is non-AI contextual/methodological evidence contrasting conventional chairside biomarker diagnostics with AI-based approaches — not part of the AI clinical-performance evidence tier. Source: Deng et al., J Periodontal Res 2022;57(4):768–779 (DOI 10.1111/jre.12999).
Tonetti et al. (2020) [20]	Prospective observational IoT monitoring study (non-ML)	N/A – MI-CLAIM not applicable (generalised mixed-effects/stepwise regression; no trained ML model)	Contextual prospective IoT monitoring study analysed with generalised mixed-effects and stepwise regression; not a trained ML model, so MI-CLAIM/APPRAISE-AI are not applicable; QUADAS-2 is also not applicable because this is not an index-test-versus-reference-	Not formally appraised with the AI/ML primary-study tools. The paper reports a prospective IoT monitoring analysis using generalised mixed-effects and stepwise regression, not a trained machine-learning model. It is therefore retained as contextual feasibility evidence for remote monitoring and excluded from the AI/ML clinical-performance tier. The reported associations should not be interpreted as externally validated ML performance; industry involvement is

Author (Year) [Ref]	Study ID / design	Tool and version (N/A with reason if not applicable)	Basis / rationale	Domain-level result, main finding, and impact on synthesis
			standard diagnostic-accuracy design	disclosed in the source. Source: Tonetti et al., J Clin Periodontol 2020;47(10):1219–1226 (DOI 10.1111/jcpe.13351).
Yan et al. (2025) [30]	Primary ML association-modelling study (non-diagnostic)	MI-CLAIM	Primary ML study quantifying an association, not a diagnostic-accuracy or classification task; QUADAS-2/APPRAISE-AI not applicable; added during revision (epub July 2025)	AI-assisted draft appraisal (pending confirmation; citation match should be reconfirmed by authors — common surname). MI-CLAIM: data partitioning partially reported with a genuine strength — true external validation against independent NHANES cohorts (AUC 0.71–0.82 across tasks, dropping to 0.72 externally); optimisation, model examination, and reproducibility not verifiable from accessible abstract-level sources. Main gap: model-development transparency unverifiable. Impact on synthesis: moderate-quality evidence, credited for genuine external validation, tempered by unconfirmed citation match and inaccessible full text. Source: likely Yan et al., J Clin Periodontol 2025;52(10):1466–1477 (DOI 10.1111/jcpe.70000).
Khubrani et al. (2025) [21]	Evidence-synthesis (systematic review/meta-analysis)	N/A – primary-study tools not applicable	Review synthesising primary studies; own methodology used APPRAISE-AI (noted in Section 2.9 for transparency, distinct from appraisal of this source as an included item)	Not formally appraised. Evidence-synthesis source; QUADAS-2/APPRAISE-AI/MI-CLAIM are primary-study tools and do not apply to a review itself. Cited as evidence-map/contextual information only; not used as clinical-performance evidence (Section 2.10 stratification).
Ferrara et al. (2025) [7]	Evidence-synthesis (systematic review)	N/A – primary-study tools not applicable	Review synthesising primary studies; own methodology used QUADAS-2 (noted in Section 2.9 for transparency, distinct from appraisal of this source as an included item)	Not formally appraised. Evidence-synthesis source; QUADAS-2/APPRAISE-AI/MI-CLAIM are primary-study tools and do not apply to a review itself. Cited as evidence-map/contextual information only; not used as clinical-performance evidence (Section 2.10 stratification).
Scott et al. (2023) [6]	Evidence-synthesis (scoping review)	N/A – primary-study tools not applicable	Review synthesising primary studies	Not formally appraised. Evidence-synthesis source. Cited as evidence-map/contextual information only; not used as clinical-performance evidence (Section 2.10 stratification).
Umer et al. (2024) [22]	Evidence-synthesis (scoping review)	N/A – primary-study tools not applicable	Review synthesising primary studies	Not formally appraised. Evidence-synthesis source. Cited as evidence-map/contextual information only; not used as clinical-performance evidence (Section 2.10 stratification).
Shuto et al. (2025) [44]	Evidence-synthesis (scoping review)	N/A – primary-study tools not applicable	Review synthesising primary studies; published 3 April 2025 (original search)	Not formally appraised. Evidence-synthesis source. Cited as evidence-map/contextual information only; not used as clinical-

Author (Year) [Ref]	Study ID / design	Tool and version (N/A with reason if not applicable)	Basis / rationale	Domain-level result, main finding, and impact on synthesis
				performance evidence (Section 2.10 stratification).
Jundaeng et al. (2024) [8]	Evidence-synthesis (narrative/scoping review)	N/A – primary-study tools not applicable	Review synthesising primary studies	Not formally appraised. Evidence-synthesis source. Cited as evidence-map/contextual information only; not used as clinical-performance evidence (Section 2.10 stratification).
Jafer et al. (2025) [9]	Evidence-synthesis (systematic review)	N/A – primary-study tools not applicable	Review synthesising primary studies; added during revision (published December 2025)	Not formally appraised. Evidence-synthesis source. Cited as evidence-map/contextual information only; not used as clinical-performance evidence (Section 2.10 stratification).
Revilla-León et al. (2023) [10]	Evidence-synthesis (systematic review)	N/A – primary-study tools not applicable	Review synthesising primary studies	Not formally appraised. Evidence-synthesis source. Cited as evidence-map/contextual information only; not used as clinical-performance evidence (Section 2.10 stratification).
Kengne Talla et al. (2024) [40]	Systematic-review protocol (teledentistry, non-AI-trial)	N/A – SPIRIT-AI not applicable	Protocol for an overview of systematic reviews on teledentistry access/quality, not an AI-intervention trial protocol; cited for the IoT/monitoring theme (Section 2.3)	Not formally appraised. Non-AI review protocol; SPIRIT-AI applies only to AI-intervention trial protocols. Cited as evidence-map/contextual information only; not used as clinical-performance evidence (Section 2.10 stratification).
Schwendicke et al. (2021) [49]	Evidence-synthesis (systematic review/meta-analysis; DL for cephalometric landmark detection – adjacent, non-periodontal-specific application)	N/A – primary-study tools not applicable	Review cited for the 'virtual patient'/multimodal-integration concept (Section 3.4), not for periodontal diagnostic performance	Not formally appraised. Evidence-synthesis source on an adjacent (non-periodontal) application. Cited as evidence-map/contextual information only; not used as clinical-performance evidence (Section 2.10 stratification).
Gusev et al. (2022) [38]	Methodological/reporting-guidance source (non-empirical)	N/A – not an empirical study	Methodological framework for ML model reporting, used to benchmark reporting standards (Section 3.5), not itself an appraisable empirical study	Not formally appraised. Methodological guidance paper, not an empirical study. Cited as evidence-map/contextual information only; not used as clinical-performance evidence (Section 2.10 stratification).
Sagatov et al. (2022) [41]	Non-AI epidemiological survey (cross-sectional)	N/A – not an AI/ML study	Population oral-health needs survey cited for Kazakhstan population context (Section 2.3), not AI evidence	Not formally appraised. Non-AI epidemiological survey; included as population-context evidence, not AI clinical-performance evidence. Cited as evidence-map/contextual information only; not used as clinical-performance evidence (Section 2.10 stratification).
Issilbayeva et al. (2025) [31]	Non-AI microbiome association study	N/A – not an AI/ML study	Oral-microbiome association study cited for local biological/population context (Section 3.7), not AI evidence; added during revision (published December 2025)	Not formally appraised. Non-AI microbiome study; included as population-context evidence, not AI clinical-performance evidence. Cited as evidence-map/contextual information only; not used as clinical-performance evidence (Section 2.10 stratification).

Author (Year) [Ref]	Study ID / design	Tool and version (N/A with reason if not applicable)	Basis / rationale	Domain-level result, main finding, and impact on synthesis
Sarakbi et al. (2025) [32]	Evidence-synthesis (scoping review)	N/A – primary-study tools not applicable	Review synthesising primary studies; added during revision (published May 2025)	Not formally appraised. Evidence-synthesis source. Cited as evidence-map/contextual information only; not used as clinical-performance evidence (Section 2.10 stratification).
Kengne Talla et al. (2023) [39]	Systematic-review protocol (teledentistry, non-AI-trial)	N/A – SPIRIT-AI not applicable	Protocol for a mixed-studies review, not an AI-intervention trial protocol	Not formally appraised. Non-AI review protocol; SPIRIT-AI applies only to AI-intervention trial protocols. Cited as evidence-map/contextual information only; not used as clinical-performance evidence (Section 2.10 stratification). Original finding retained: Framework of barriers to teledentistry implementation

Abbreviations: AI, artificial intelligence; APPRAISE-AI, checklist for AI diagnostic-accuracy study appraisal; CONSORT-AI, extension of the CONSORT statement for randomised trials evaluating AI interventions; DL, deep learning; LMIC, low- and middle-income country; MI-CLAIM, Minimum Information About Clinical Artificial Intelligence Modelling checklist; ML, machine learning; QUADAS-2, Quality Assessment of Diagnostic Accuracy Studies 2; SPIRIT-AI, extension of the SPIRIT statement for protocols of trials evaluating AI interventions.
